# Assessment of suitable cultivation area for *Paris polyphylla* var. *chinensis* and var. *yunnanensis* under anthropogenic disturbance based on ensemble modeling and germplasm identification

**DOI:** 10.1186/s12870-025-08010-7

**Published:** 2026-01-14

**Authors:** Yiheng Wang, Hangxiu Liu, Dan Zhao, Sheng Wang, Jingyi Wang, Xiulian Chi, Chengcai Zhang, Tielin Wang, Chaogeng Lyu, Chuanzhi Kang, Jiahui Sun, Lanping Guo, Luqi Huang

**Affiliations:** 1 State Key Laboratory for Quality Ensurance and Sustainable Use of Dao-di Herbs, National Resource Center for Chinese Materia Medica, China Academy of Chinese Medical Sciences, Beijing, 100700 China; 2 Key Laboratory of Biology and Cultivation of Herb Medicine, Ministry of Agriculture and Rural Affairs, Beijing, 100700 China; 3 Dexing Research and Training Center of Chinese Medical Sciences, China Academy of Chinese Medical Sciences, Dexing, 334220 China; 4 Faculty of Life Science and Technology, Kunming University of Science and Technology, Kunming, 650500 China; 5 Institute for ecology of Chinese materia medica resources, Deqing, 313200 China; 6 China Academy of Chinese Medical Sciences, Beijing, 100700 China

**Keywords:** Suitable distribution, *Paris chinensis*, *Paris yunnanensis*, Anthropogenic impacts, Ensemble modeling, Mini-barcodes

## Abstract

**Background:**

Rhizoma Paridis, a medicinal plant material with significant economic value, is derived from two species: *Paris polyphylla* var. *chinensis* (Pc) and var. *yunnanensis* (Py). However, the challenge in accurately distinguishing these two morphologically similar taxa, combined with uncertainty regarding their optimal cultivation zones under changing environmental conditions, creates significant obstacles for sustainable cultivation and quality control of this valuable medicinal resource. Climate change and human activities are major factors influencing suitable habitats for these species. Under different greenhouse gas emission scenarios (SSP1-2.6 and SSP5-8.5 representing low and high emissions respectively), climate impacts on species distributions will vary significantly. To address these challenges and support evidence-based conservation and cultivation strategies, this study utilized an ensemble forecasting machine learning approach to model current and future distribution scenarios under varying levels of greenhouse gas emissions. Comparative genomic techniques for species-specific barcoding were employed to analyze the impacts of anthropogenic activity and climatic changes on the distributions of the plants and species identification.

**Results:**

Our findings indicated that the ecological niches of Pc and Py varied significantly: Pc is predominantly suited to southern China, while Py would thrive in Yunnan Province. Annual precipitation was the principal determinant of the distribution of Pc, whereas the annual temperature range held greater significance for Py. Human activity exerts a more substantial impact on the distribution of Pc than on Py. Under low-emission scenarios, Pc is projected to significantly expand its ultrahigh-suitability range by the end of the century, whereas the range of Py will remain relatively stable. Conversely, under high emissions, the distribution of Pc would initially expand before contracting, and that of Py would significantly decline. These shifts highlight the critical importance of maintaining low emissions for species conservation and sustainable use and help us delineate long-term cultivation areas. Our newly developed mini-barcodes were effective in distinguishing between Pc and Py.

**Conclusions:**

This comprehensive analysis provides essential insights for the sustainable cultivation and optimized yield of the two plant species, thereby providing a foundation for enhanced economic returns.

**Supplementary Information:**

The online version contains supplementary material available at 10.1186/s12870-025-08010-7.

## Introduction

The distributions of plants are primarily determined by climate. Over the past century, global temperatures have risen by nearly 1 °C, with the most rapid increases occurring in the last 30 years [1]. This temperature rise is a clear manifestation of the impact of human activities such as carbon emissions on the climate. Some regions have experienced more severe warming, with a temperature increase of 0.2 °C per decade [2]. Anthropogenic activities such as extensive land use causing habitat loss have also had direct effects on large-scale range contraction and/or expansion of many species [3]. Shifts in distributions have profound effects on the types and quantities of plant secondary metabolites that are the chemical foundation of herbal medicines [4, 5].

According to the Chinese Pharmacopoeia, Paridis refers to the dried rhizome of the perennial herbs *Paris polyphylla* var. *yunnanensis* (Py), restricted to Yunnan, and *P. polyphylla* var. *chinensis* (Pc). This herbal remedy is a well-known traditional medicinal material with a long history of use in China. The material from Py is considered better than that from Pc [6]. The current market prices are ¥200/Kg for Py and ¥160/Kg for Pc (https://www.zyctd.com/). However, the sustainability of this valuable resource faces critical challenges. As an important understory species in forest ecosystems, it also plays a crucial ecological role in maintaining biodiversity and ecosystem stability [7, 8]. Despite its ecological importance, wild populations are under severe pressure from anthropogenic disturbances including deforestation, agricultural expansion, and particularly unsustainable wild collection driven by high commercial demand [9]. As an important understory species in forest ecosystems, it also plays a crucial ecological role in maintaining biodiversity and ecosystem stability [7, 8]. These mounting pressures have resulted in these species being listed as “Vulnerable” on the IUCN Red List, with populations experiencing continuous decline due to overexploitation, habitat degradation, and illegal collection for medicinal trade [10, 11]. Consequently, sustainable cultivation has become essential to meet medicinal demands while protecting wild populations, making the prediction of optimal cultivation zones a research priority.

For good medicinal efficacy, the plants must be grown for six to seven years, a situation that entails a high investment risk when selecting Py or Pc and the location of plantations. The extended cultivation period amplifies the consequences of incorrect germplasm selection or unsuitable site placement, making accurate species identification and precise habitat prediction crucial for sustainable cultivation success. Py and Pc can only be discriminated morphologically by their inner tepals, as their seeds, seedlings, and rhizomes are indistinguishable (Fig. S1A). This morphological similarity creates significant challenges for cultivators, as misidentification during the seedling stage can result in years of inappropriate cultivation practices, ultimately leading to substandard medicinal quality and economic losses [6].

To address these identification challenges, DNA barcoding has emerged as the most reliable solution for accurate germplasm identification [12]. Universal DNA barcodes such as *matK*, *rbcL*, *trnH-psbA*, and *ycf1* (from plastid genomes) and ITS (from nuclear genomes) have been widely employed in the species identification of plant materials [13–16]. Due to the limited lengths and medium to low polymorphism of the plastid fragments, the identification sometimes fails to discriminate between closely related species; in such cases, DNA superbarcodes, i.e., whole sequences of plastid genomes, can be employed [17–19]. Although conventional DNA barcodes are fragments 400 ~ 600 bp in length, PCR amplification of these DNA fragments from processed medicinal materials remains challenging [20, 21]. Therefore, DNA mini-barcodes, i.e., shorter segments ranging from 100 to 250 bp, are sometimes used [22]. DNA mini-barcodes are generally developed by comparing whole plastomes and selecting regions of high polymorphism. The plastid DNA mini-barcodes can be concatenated, and they often demonstrate excellent powers of discrimination. This strategy has been successfully applied to the development of DNA mini-barcodes for many medicinal plants, including *Panax*, *Coptis*, and *Ziziphus* [21, 23, 24].

Equally critical to cultivation success is the accurate prediction of suitable growing environments, particularly under changing climatic conditions and human influence. Species distribution models (SDMs) provide the necessary framework for linking species distributions with environmental/human factors and predicting optimal cultivation zones [25–27]. In previous studies, single models were applied to predict species distributions; these included the maximum entropy model (Maxent) [28], generalized linear models (GLM) [29], multiple adaptive regression splines (MARS) [30], surface distance envelope (SRE) [31], artificial neural networks (ANN) [32], and random forest (RF) [33]. However, individual SDMs exhibit substantial variation in prediction results and performance, with no single model consistently outperforming others across different species and environmental factors [34]. To address these limitations, ensemble modeling has emerged as a superior approach that combines predictions from multiple algorithms, thereby significantly improving prediction robustness and reducing model uncertainty while enhancing overall accuracy [25, 35]. The ensemble approach is particularly justified for long-term cultivation planning, as it provides confidence intervals and uncertainty estimates essential for risk assessment in agricultural investment decisions. Currently, the ensemble modeling (EM) R package “biomod2” is used to construct an EM for distribution prediction [36–38]. The R package “ecospat” further complements this by visualizing ecological niche dynamics under varying scenarios such as the influence of human activity [39]. This EM technique offers a comprehensive framework for more accurate and robust ecological analyses.

Climatic change, germplasm (variety) differences, and human activities profoundly influence every aspect of herbal medicine cultivation, from growing and harvesting to processing, ultimately shaping the quality of the herbs. Selecting appropriate cultivation sites for given germplasms is especially critical for species with long cultivation periods. This study focuses on the taxonomic status of Pc and Py based on plastid genomics, and we use EM to estimate their potential distributions. The objectives of the study are (1) to determine whether Pc and Py are two species or two varieties of a single species and develop mini-barcodes for their discrimination; (2) to quantify the effects of human activity on the ecological niches of Pc and Py, and (3) to infer these plants’ future distributions and dynamics under different shared socio-economic pathways (SSPs). The results will be helpful for the establishment of long-term cultivation sites that will be able to withstand future climate change and ensure the quality of Rhizoma Paridis derived from Pc and Py.

## Materials and methods

### Plant material and DNA extraction

Three Pc accessions were collected in China during the Fourth National Survey of Chinese Materia Medica Resources for novel plastome sequencing. The voucher specimens were identified by Prof. Jiahui Sun and deposited at the herbarium of the National Survey of Chinese Materia Medica Resources (Daxing District, Beijing, China) with the voucher specimens numbers 520325140529165LY, 520325160427568LY and 422802120614011LY. These newly sequenced plastomes were combined with other five Pc and twelve Py accessions downloaded from GenBank formed dataset I for barcodes development. Dataset II comprised 61 plastome sequences, including 41 sequences of other *Paris* species and *Trillium* species (used as outgroups) obtained from GenBank, together with the 20 sequences from dataset (I) This combined dataset was used for phylogenomic analysis. To further clarify the divergence time between Pc and Py, we constructed dataset III for molecular dating by selecting one representative sequence from each species in dataset (II) All samples mentioned above, including samples downloaded from GenBank, and self-collected in this study, were listed in Table S1 with their corresponding dataset information. DNA mini-barcodes were developed based on the plastid genome sequences of Pc and Py. A collection of 42 seedling samples, comprising 18 for Pc and 24 for Py (Table S2), was sourced from vendors to test the discriminatory power of the newly developed DNA mini-barcodes. Total DNA was extracted using a modified cetyltrimethylammonium bromide (mCTAB) method [40].

### Plastome sequencing, assembly, and annotation

The purified DNA was sonicated into 300–350 bp fragments, and paired-end sequencing libraries were constructed using a NEBNext UltraTM DNA library prep kit (New England Biolabs, Ipswich, MA, USA). PE150 sequencing was performed on the Illumina HiSeq XTen platform at Novogene Co., Ltd (Beijing, China). Fastp 0.23.2 software was used to filter the raw data, generating high-quality clean data for plastid genome assembly under the default settings [41]. GetOrganelle v1.7.7 software was employed for de novo plastome assembly with the following settings: -F embplant_pt, -R 15, and -k 85,105 [42]. Plastome annotation was carried out using the tool CPGAVAS2, with “2544 plastomes” as a reference dataset, and manually checked for missing or incorrectly annotated genes in Sequin [43]. The annotation results were further checked using Geneious v8.1.

### Plastid phylogenomics and estimation of divergence time

Plastome sequences of dataset I were aligned using MAFFT online and manually verified with MEGA v7 [44]. The number of indels and parsimony-informative sites (Pi) was calculated in each 500-bp sliding window using DnaSP v5.10. To illustrate the plastome hotspot locations, a circos plot was created using the “OmicStudio” online platform (https://www.omicstudio.cn/tool/) using the Pi data [45]. Variety-specific variants were manually enumerated with MEGA v7 for mini-barcode development based on SNPs and indels. Primers for amplifying DNA mini-barcodes were designed and synthesized at Sangon Biotech (Shanghai, China). The quality and size of the PCR products were assessed using 2% (w/v) agarose gels. The fragments of DNA mini-barcodes were sequenced at Sangon Biotech, and the R package “ggmsa” was used to visualize the Sanger sequencing results of variety-specific mini-barcodes [46].

Sequences of dataset II for phylogenomics were aligned using the MAFFT and ambiguous regions were trimmed by the Gblocks 0.91b program [47]. The maximum likelihood tree was inferred using IQ-TREE with the TVM + F + I + R4 model and 5,000 ultrafast bootstraps in PhyloSuite [48, 49]. The dataset III was used for dating analysis. BEAST v2.6.6 was employed with the following parameter settings: a Relaxed Log Normal clock model, a Yule speciation model, and a GTR substitution model [50]. Given the absence of a reliable calibration fossil for the genus *Paris*, the divergence times of the genus *Trillium* (32.11 Mya [51]) and the clade comprising *P. tetraphylla*, *P. verticillata*, and *P. incompleta* (10.93 Mya [52]) were used as secondary calibration points. The Markov chain Monte Carlo (MCMC) chains were run for 400 million generations, with samples taken every 10,000 generations. Effective sample sizes (ESSs) were verified using Tracer v1.6 to ensure all parameters were above 200. The first 25% of runs were discarded as a burn-in, and TreeAnnotator v2.6.6 (BEAST package) was used to produce a maximum clade credibility (MCC) tree. Divergence times and 95% highest posterior density (HPD) intervals were visualized with FigTree v1.3.1 and refined using Adobe Illustrator CS6.

### Occurrence records and preprocessing of variables

To analyze the distributions of Pc and Py in China, occurrence records were obtained from the Global Biodiversity Information Facility (GBIF; https://www.gbif.org/) and the National Plant Specimen Resource Center (NSII; http://www.nsii.org.cn/). For occurrence record quality assurance, the authors set the distribution maps for each species in ArcGIS 10.8, checked them manually, and eliminated any uncertain spots. To reduce sampling bias, spatial rarefaction was implemented at a 10-km resolution using SDMtoolbox v2.5, resulting in 199 records for Pc and 109 records for Py [53].

We extracted current climatic variables from the WorldClim 2.1 database (https://www.worldclim.org/) at a 2.5′ resolution, including 19 bioclimatic variables (bio1 to bio19) and two future periods (2050s: 2041–2060, 2090s: 2081–2100) based on the BCC-CSM2-MR model. SSP126, representing strict greenhouse gas controls with a 2.6 W/m² forcing by 2100, and SSP585, indicating high greenhouse gas concentrations under an absence of climate change mitigation policies, leading to an 8.5 W/m² forcing by 2100. Elevation data were downloaded from the EarthEnv database (https://www.earthenv.org/) [54]. There are three closely related factors that can be used to quantify human activity: the Human Footprint Index (HFI), the Human Population Density (HPD), and the proportion of cropland [3]. To quantify the influence of human activity on the distributions of Pc and Py, the HFI map (the 2009 Human Footprint, Last of the Wild Project, Version 3, 2018 Release [55]) was chosen as a variable to model the plants’ distributions with and without human influence under the current climate situation. The HFI map measures cumulative human pressure on a scale from 0 to 50 using eight variables representing human settlement (population density, built-up environments, electric power infrastructure), access (roads, railways, navigable waterways), and landscape transformation (croplands, pasture lands) [56]. To ensure accuracy and reduce overfitting, a Pearson correlation analysis of the data for 19 climatic variables extracted based on rarefied records was performed using the R package “ggpairs.” After deleting correlation pairs with |r| > 0.7, six climatic variables remained (Table S3). These, along with elevation and HFI, were used for the simulations of suitable habitat. The OmicStudio platform was employed for a comparative analysis of these environmental factors [45]. Furthermore, using mean annual precipitation and mean annual temperature data from 1970 to 2000, the Whittaker biome classification for Pc and Py was computed with the R package “plotbiomes” to enhance our understanding of their ecological niches [57].

### Construction of SDM and evaluation of model accuracy

Our modeling approach assumed that: (1) climate change and human activities are the primary drivers of species distribution changes [58, 59]; (2) species respond to climate change primarily through range shifts rather than rapid evolutionary adaptation within the several-decade timeframe [60, 61]. Based on these assumptions, the modeling of suitable distributions for Pc and Py with/without human influence and of the potential future distributional dynamics was performed using the R package “biomod2” [62]. We used twelve different algorithms implemented in “biomod2”: GLM, GBM, GAM, CTA, ANN, SRE, FDA, MARS, RF, Maxent, Maxnet, and XGBoost. Model calibration and validation followed a robust framework: For each modeling approach, 75% of the distribution records were randomly selected for model calibration (training), and the remaining 25% were reserved for independent validation (testing). To ensure robust performance assessment and reduce potential bias from single data splits, each model was run with 5 replications. To better simulate the actual distributions and reduce spatial deviation, 1,000 pseudo-absence points were randomly generated using a random sampling strategy, and this process was repeated five times to provide comprehensive background data for model calibration. This approach ensures unbiased model evaluation by testing performance on independent validation data that remained separate from the calibration process.

ROC and TSS were selected to evaluate the single model performance. ROC values range from 0 to 1, where values above 0.9 indicate excellent performance, and TSS value ranges from − 1 to 1, where values above 0.75 indicate excellent model performance [63–65]. Models that met the criteria of a TSS exceeding 0.8 and a ROC value exceeding 0.9 were selected for further EM prediction. Detailed single model information for ensemble prediction used were listed in Table S4. The EM results provided the probability of presence values from 0 to 1 that were classified into five suitability grades using an equal interval approach in ArcGIS 10.8: no suitability (0-0.2), low suitability (0.2–0.4), medium suitability (0.4–0.6), high suitability (0.6–0.8), and ultrahigh suitability (0.8-1). The key environmental variables were identified using the code snippet “get_variables_importance(myBiomodModelOut)” in the biomod2 R package. Additionally, all future habitat projections were based on current results under the influence of human activity.

### Migration of potential geographical distribution centers and measurement of ecological niche overlap

The potential geographical distribution centers of Pc and Py, considering both anthropogenic influence and natural conditions across various time periods and climate scenarios, were calculated using SDMtoolbox v2.5 integrated with ArcGIS 10.8. Migration tracks were visualized by comparing distribution centers across these scenarios. To assess the changes in distribution areas, the “Distribution changes between binary SDMs” tool in SDMtoolbox v2.5 was employed using a suitability threshold of > 0.6. The overlapping geographical distribution analysis performed in ArcGIS 10.8 was used to locate long-term suitable planting regions for Pc and Py, both individually and together, under different climate scenarios. First, we classified potential areas of suitability equal to or higher than 0.6 as “suitable” and those lower than 0.6 as “unsuitable.” Then, we superimposed the layers from different periods of Pc and Py and ultimately retained the areas that showed suitability in all periods as the long-term suitable planting regions.

In this study, we used the changes in ecological niches to quantify the extent of anthropogenic impact on species. Ecological niche dynamics based on occurrence and bioclimatic data were compared between areas with and without anthropogenic influence (whether or not to add an HFI layer) using the R package “ecospat”. Ecological niche differentiation was analyzed using PCA-env approach in ENMTools [66]. Niche overlap was quantified using Schoener*’s D* with statistical significance tested through niche equivalency tests (*n* = 1000 iterations) [67]. *D* ranges from 0 to 1, indicating no overlap to the total overlap of ecological niches. A value of *D* > 0.6 indicates significant overlap, and the deviation from 1 can be used to infer the impact of human activity on species distributions [26].

## Results

### The relationship between Pc and Py

**Fig. 1:**
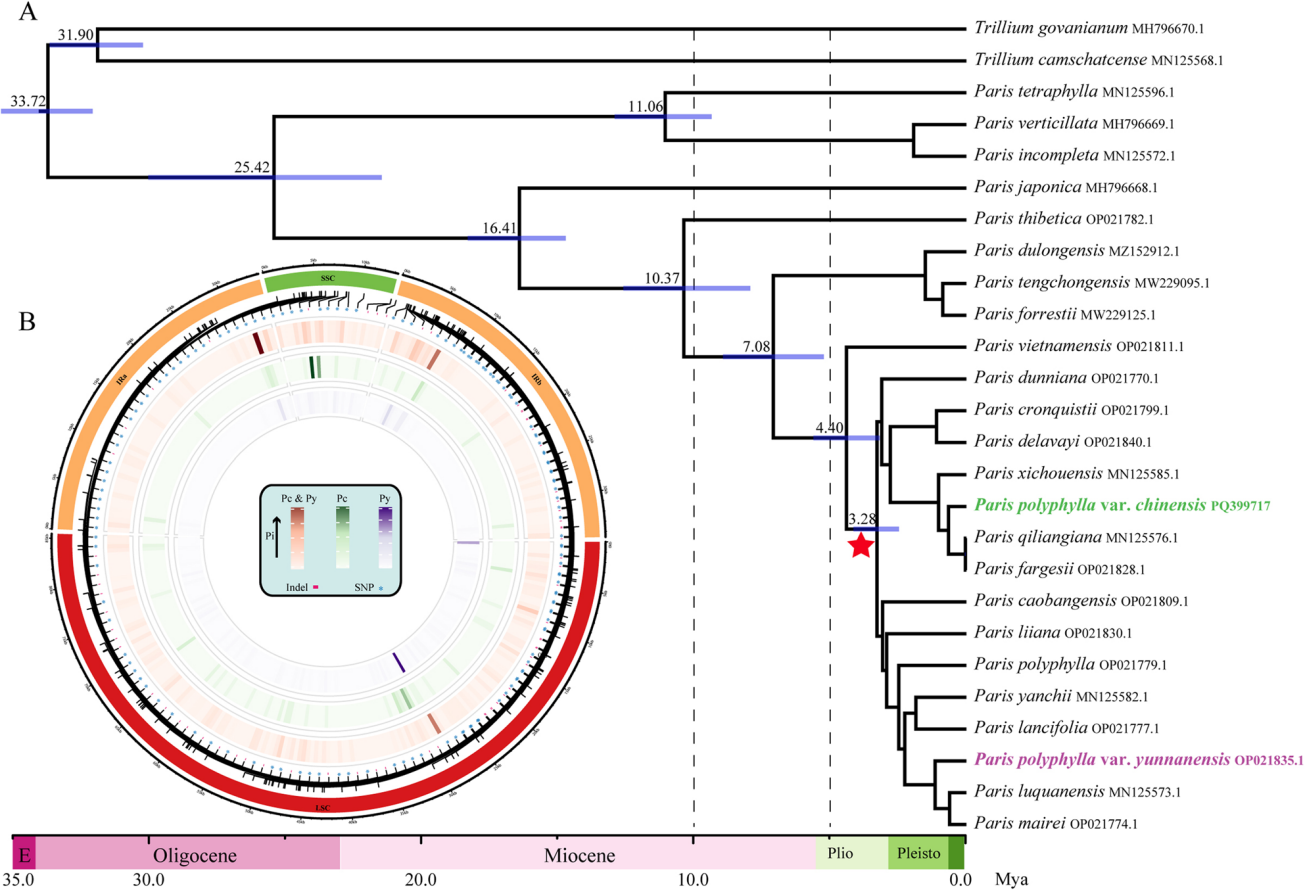
Estimation of divergence time for Pc and Py (**A**, generated using dataset III) and their genetic variation (**B**, generated using dataset I)

The phylogeny based on complete plastid genomes neither indicated that Pc and Py were conspecifics nor suggested a close relationship between Pc and Py (Fig. 1 and Fig. S2). Instead, the results indicated their belonging to different lineages, and that they should be considered as distinct species, i.e., as *P. chinensis* (Pc) and *P. yunnanensis* (Py). The divergence time between Pc and Py was estimated to be approximately 3.28 million years ago (Ma) in the late Pliocene (Fig. 1A), much longer than some of their congeners. Genetic variants were predominantly located in the large single-copy (LSC) region, small single-copy (SSC) region, and the inverted repeat (IR) boundary areas. Given such pronounced species divergence, we scanned and compared dataset I of the plastome data comprising eight Pc and twelve Py accessions and obtained 202 species-specific loci including SNP and Indels for the development of mini-barcodes (Table S5). To demonstrate the feasibility of barcode development method and enhance the germplasm identification of Pc and Py, we randomly chose two SNP site to develop mini-barcodes with target fragment lengths of 200 bp. These two species-specific barcodes were verified using a set of 42 seedling samples (eighteen Pc and 24 Py) obtained from vendors with guaranteed seedlings. Our newly developed markers had excellent amplification and identification success rates, and their primer sequences are currently under patent application. For convenience, we have shown only five accessions of each species with sequence data and specific sites in Fig. S1B. These genetic findings and molecular tools offer a robust framework for the accurate identification of Pc and Py, supporting conservation efforts and authentication of herbal products.

### Model evaluation and key climatic variables

Under stringent model performance criteria (TSS > 0.8 and ROC > 0.9), six algorithms—CTA, GAM, GBM, MARS, RF, and XGBoost—were identified as effective for modeling the distribution of Pc with and without human influence. These were further utilized for EM predictions. For Py, all algorithms except SRE met the criteria and were incorporated into the EM process (Fig. S3). The integration of these robust algorithms ensured the high reliability of the simulation outcomes.

**Fig. 2:**
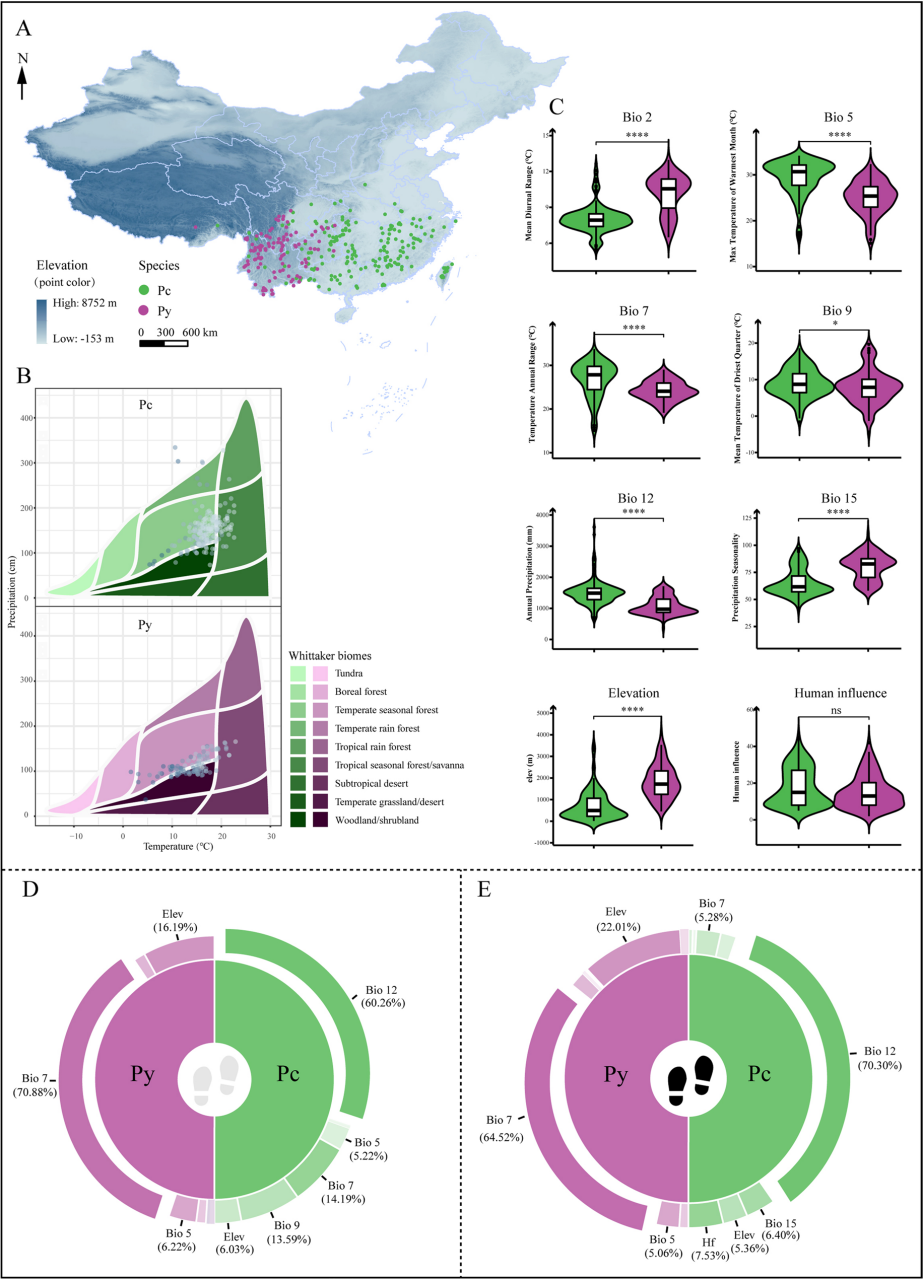
Distributional features of Pc and Py and dominant climatic factors affecting the distributions. Pc is shown in green, and Py is shown in purple. **A **Rarefied occurrence record distribution. **B** Whittaker biome plot of occurrence records showing the mean annual temperature (°C) and mean annual precipitations (cm) for each species. **C** Violin plots for environmental factor comparisons, * indicates *P* < 0.05; **** indicates *P* < 0.001; ns indicates not significant. Additionally, the contribution rates of environmental factors influencing suitable distributions of Py and Pc without (**D**) or with (**E**) anthropogenic impacts are shown. The dominant variable factor has been marked for emphasis

We plotted the rarefied occurrence of Pc and Py on a map of China, with Pc spread across the Yangtze River basin and southern China, and Py more concentrated in southwest China (Fig. 2A). According to the Whittaker biome classification, Pc and Py occurred in distinct biomes, with Pc being more widespread, predominantly in temperate seasonal forests and secondarily in tropical seasonal forests and woodlands/shrublands. In contrast, Py had a more centralized distribution, primarily in temperate seasonal forests and woodlands/shrublands (Fig. 2B). Meanwhile, between-species differences in the six climatic variables screened by the correlation analysis and the elevation and human activity factors were compared. The two species differed significantly in Bio 2, Bio 5, Bio 7, Bio 12, Bio 15, and elevation (Fig. 2C), indicating that they occupy distinct ecological niches. Despite these differences, there was no significant difference in exposure to anthropogenic impacts between the species. Bio 12 (annual precipitation) was the dominant factor influencing Pc distribution, contributing 70.30% with and 60.26% without human impact. In contrast, Bio 7 (temperature annual range) was the most important factor for Py, with a contribution rate of 64.52% with and 70.88% without human influence (Figs. 2D & E, Table 1).

**Table 1:** Importance contribution rate of each environmental variable for P. c and P. y based on EM

Pc		Pc with human influence		Py		Py with human influence	
Environmental variables	Contribution (%)	Environmental variables	Contribution (%)	Environmental variables	Contribution (%)	Environmental variables	Contribution (%)
Bio 2	0.366	Bio 2	0.91	Bio 2	2.15	Bio 2	1.96
Bio 5	5.22	Bio 5	0.799	Bio 5	6.22	Bio 5	5.06
Bio 7	14.2	Bio 7	5.28	Bio 7	70.9	Bio 7	64.5
Bio 9	13.6	Bio 9	3.42	Bio 9	2.65	Bio 9	3.41
Bio 12	60.3	Bio 12	70.3	Bio 12	1.13	Bio 12	0.858
Bio 15	0.345	Bio 15	6.4	Bio 15	0.778	Bio 15	0.378
Elev	6.03	Elev	5.36	Elev	16.2	Elev	22
		Human Footprint	7.53			Human Footprint	1.8

### Current potential distribution and changes caused by human activity

**Fig. 3:**
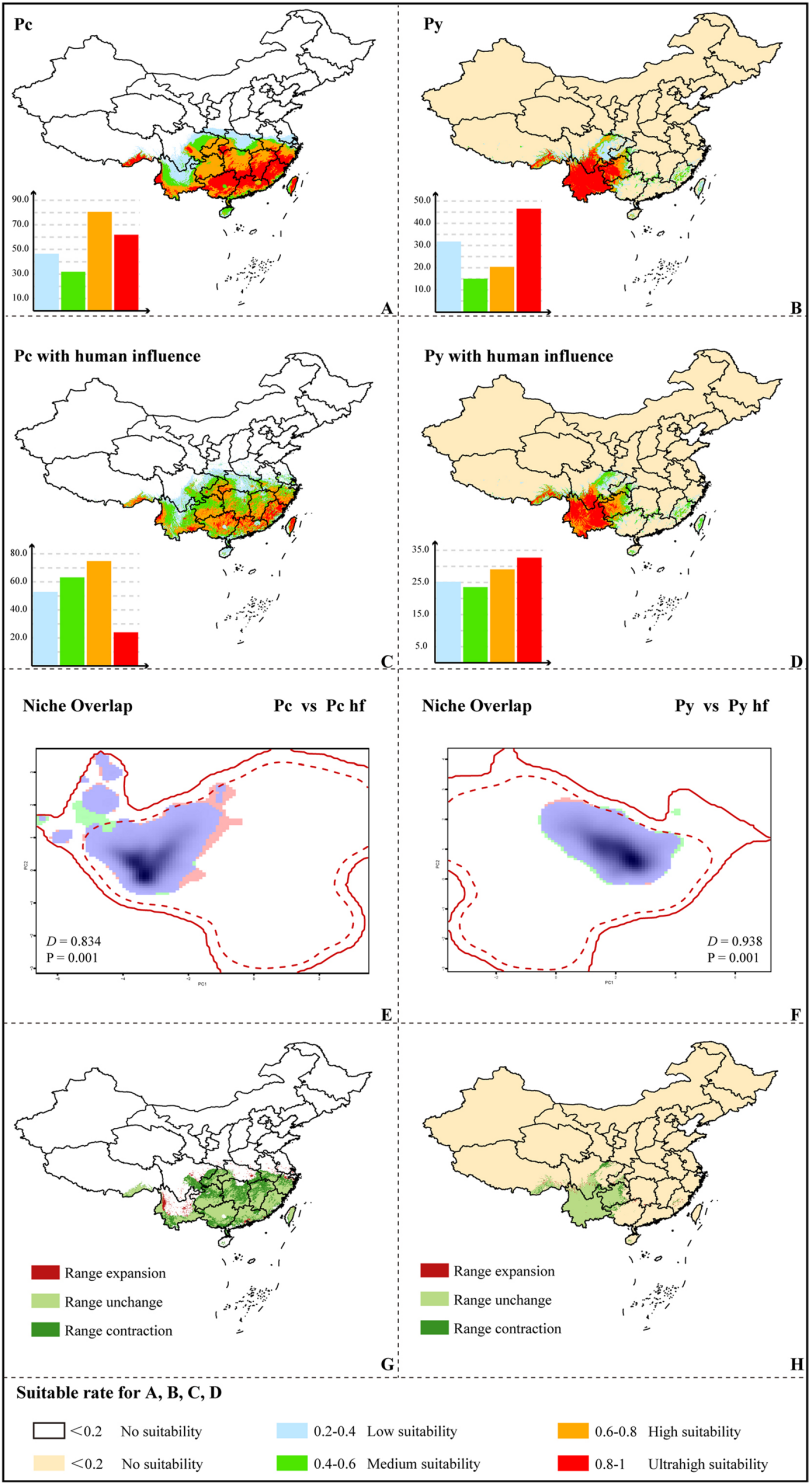
Shifts in suitable cultivation areas and differences in niche overlap between Pc and Py under anthropogenic impacts. The suitable cultivation areas of Pc without (**A**) or with (**C**) anthropogenic impacts and Py without (**B**) or with (**D**) anthropogenic impacts are shown. Five levels of suitability are shown in different colors as follows: no suitability (0-0.2, white or flesh color); low suitability (0.2–0.4, blue); medium suitability (0.4–0.6, green); high suitability (0.6–0.8, yellow); ultrahigh suitability (0.8-1.0, red). The histograms represent the area corresponding to each suitability level, and the vertical coordinate units are ×10^4^ km^2^. Changes in ecological niches pre- and post-anthropogenic impact for Pc (**E**) and Py (**F**) are shown. The green and pink shadings represent densities of species occurrences in the presence or absence of anthropogenic impact; blue represents overlap. The shifts of suitable cultivation areas between the presence or absence of anthropogenic impact scenarios under suitability conditions > 0.6 are shown for Pc (**G**) and Py (**H**)

Based on the EM projections, the current potential distribution (suitability rate > 0.2) of Pc covers an area of 2.20 × 10^6^ km^2^ (46.47 × 10^4^, 30.10 × 10^4^, 80.59 × 10^4^, and 61.97 × 10^4^ km^2^, respectively, for suitability of 0.2–0.4, 0.4–0.6, 0.6–0.8, and 0.8-1, Fig. 3, Table S6). The current potential distribution area is more restricted for Py, with a coverage of 113.79 × 10^4^ km^2^ (31.78 × 10^4^, 15.05 × 10^4^, 20.38 × 10^4^, and 46.58 × 10^4^ km^2^, respectively, for suitability of 0.2–0.4, 0.4–0.6, 0.6–0.8, and 0.8-1, Fig. 3). The ultrahigh-suitability regions for Pc were primarily located in Guangxi, Guangdong, Fujian, Jiangxi, and Zhejiang provinces. In contrast, those of Py were concentrated in Yunnan Province, with minor occurrences in Sichuan and Guizhou provinces. When superimposed on anthropogenic impacts, the potential distributions of the two species were slightly reduced, at 214.74 × 10^4^ km^2^ for Pc and 110.47 × 10^4^ km^2^ for Py. For Pc, the ultrahigh-suitability area declined significantly to 23.94 × 10⁴ km², while that of Py experienced a moderate reduction to 32.68 × 10⁴ km², highlighting the pronounced effect of human activity on Pc. The impact of human activity on the distribution of Pc was more substantial, with the contribution rate of human activity accounting for 7.5%, much higher than the 1.8% observed for Py (Fig. 2E; Table 1). A similar phenomenon was indicated by the ecological niche-overlap analysis. Pc deviated from *D* = 1 to a greater extent than Py, with the obtained *D* values of 0.834 for Pc and 0.938 for Py indicating that human impact was much higher for Pc than for Py (Figs. 3E & F). The expansion and contraction analysis in high- and ultrahigh-suitability areas (suitability rate > 0.6) showed that Pc had a retention rate of 69% under the influence of human activity. More severe contraction was revealed in the Yangtze River basin for Pc, probably due to the urbanization in this region, while slight expansion was indicated in the region of the Nushan Mountain Range in western Yunnan. In contrast, Py retained over 90% of its highly suitable area, indicating relatively minor anthropogenic impacts. There was a substantial ecological niche difference between Pc and Py, with a small estimated niche overlap of *D* = 0.285. Principal component analysis captured 81.93% of environmental variance (PC1: 55.96%, PC2: 25.97%, Fig S4B), with Bio5, Bio7 and elevation identified as the primary drivers of niche differentiation. The equivalency test strongly rejected niche equivalency (p value = 1, Fig S4C) underscoring the species’ distinct environmental preferences (Fig. S4).

### Dynamic changes in suitable habitat under different climate scenarios

**Fig. 4:**
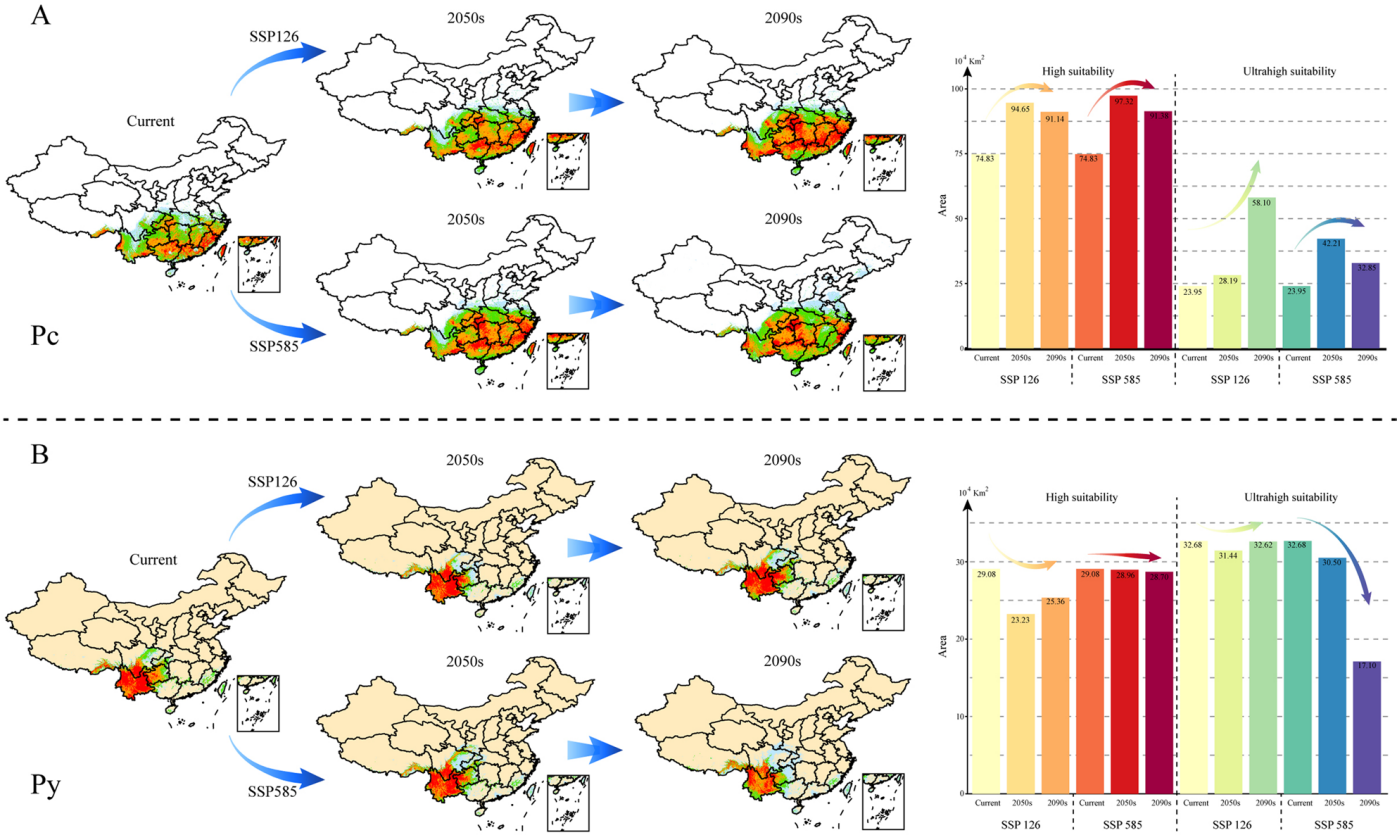
The dynamic changes in the suitable cultivation areas of Pc (**A**) and Py (**B**) in the future under two greenhouse gas emission scenarios (SSP126 and SSP585). The histograms indicate the change in area for high suitability and ultrahigh suitability under different emission models, respectively

Two climate-change scenarios, SSP126 (low greenhouse gas emissions) and SSP585 (high greenhouse gas emissions) were analyzed to predict the suitable habitats of Pc and Py in the 2050s and 2090s (Fig. 4, Table S6). Pc showed an upward tendency followed by a downward trend under both climate scenarios, with the peak occurring at 94.65 × 10^4^ km^2^ in the 2050s under SSP126 and 97.32 × 10^4^ km^2^ under SSP585. For Py, there was a downward trend followed by an upward trend, with the smallest distribution occurring at 23.23 × 10^4^ km^2^ in the 2050s under SSP126, and then a slow decline, dropping to 28.70 × 10^4^ km^2^ in the 2090s under SSP585. More drastic changes were estimated for both varieties in the ultrahigh-suitability category. The ultrahigh-suitability area for Pc sharply increased to reach 58.10 × 10^4^ km^2^ in the 2090s under the SSP126 scenario, while that of Py fell to 17.10 × 10^4^ km^2^ in the 2090s under the SSP585 scenario.

### Potential geographical distribution center shift and long-term suitable areas

Under the combined influence of human activity and future greenhouse gas emissions, the distribution centers of Pc and Py are projected to shift differently across scenarios (Fig. S5). For Pc, the center of potential geographical distribution is expected to migrate northeast by the 2050s, then shift northwest by the 2090s. Under low (SSP126) and high-emission (SSP585) scenarios, its center will move from the current Xinning County (Hunan-Guangxi border) to Dongkou County (Hunan Province) and Hongjiang City (Hunan-Guizhou border) by the 2090s, respectively. For Py, the distribution center is projected to remain primarily on the Yunnan-Sichuan border, and to migrate from Wuding County (Yunnan) to Huili City (Sichuan) and to Yongren County (Yunnan) by the 2090s under SSP126 and SSP585, respectively.

**Fig. 5:**
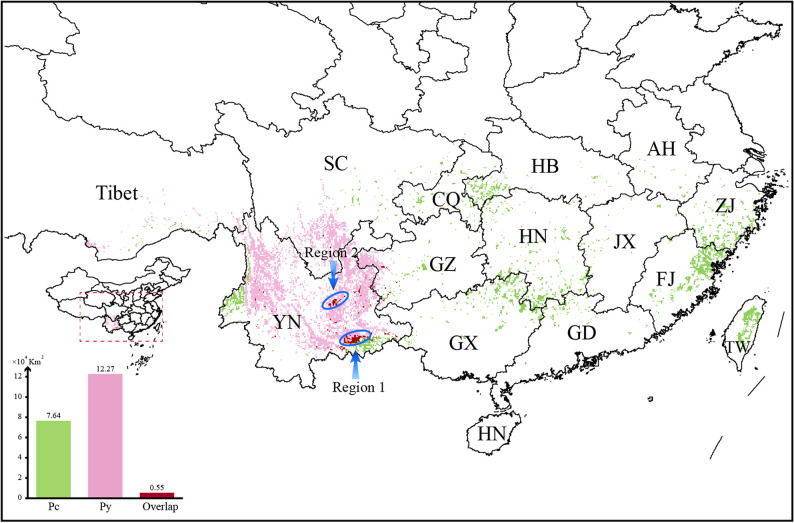
Long-term cultivation areas for Pc and Py individually, and for both species. SC, Sichuan; YN, Yunnan; CQ, Chongqing; GZ, Guizhou; GX, Guangxi; HB, Hubei; HN, Hunan; GD, Guangdong; AH, Anhui; JX, Jiangxi; ZJ, Zhejiang; FJ, Fujian

We intersected areas with current and future suitability rates greater than 0.6 in different time periods and emission conditions as the long-term suitable sites for cultivation or germplasm nursery establishment. Such regions were assumed to be suitable for cultivation of the corresponding species for a hundred years into the future, regardless of greenhouse gas emission scenarios. The long-term suitable area of Pc was calculated to be about 7.64 × 10^4^ km^2^, dispersed across South China, with a higher concentration in northern Fujian Province. The long-term suitable area of Py was larger, about 12.27 × 10^4^ km^2^, and concentrated in Yunnan and southern Sichuan. Meanwhile, two regions in Yunnan, with a total area of about 0.55 × 10^4^ km^2^, were found to be suitable for long-term cultivation of both species (Fig. 5).

## Discussion

### Pc and Py are distinct species

Pc and Py are treated as two varieties of *P. polyphylla*, together with another six *P. polyphylla* varieties, in the Flora Reipublicae Popularis Sinicae (FRPS), a convention that was followed by the Chinese Pharmacopoeia; this may have been due to *Paris* having been insufficiently studied at the time of publication. Recent publications have overturned the previous taxonomy of the genus, finding that Pc and Py are not closely related phylogenetically [18, 52, 68]. The taxa deserve distinct species status as P. chinensis (Pc) and P. yunnanensis (Py) despite their very similar morphology. Our findings support these previous phylogenetic studies and further demonstrate significant ecological niche differentiation between the two species (Schoener’s *D* = 0.285, p-value = 1), providing additional evidence for their taxonomic separation. Although upgrading the taxonomic rank of Pc and Py to species level does not affect the data extracted based on varietal rank, it more accurately reflects their degree of relatedness.

Although Pc and Py can be discriminated by stubby (Pc) versus slender (Py) peduncles, inner tepal characteristics and length and other traits, the seeds and seedlings of Pc and Py are indistinguishable in the market. Medicinal plants with long circulation cycles must be grown in the proper environment to develop their highly active medicinal components [69]. There is an urgent need for efficient and reliable methods by which to identify seeds, seedlings, and even processed medicines. High-performance liquid chromatography (HPLC), near-infrared spectroscopy (NIR), and DNA barcoding with ITS2 have been employed to differentiate species of *Paris* [18, 70–72]. However, these methods are not sufficiently accurate or convenient, especially for processed medicines. The plastid mini-barcodes developed in this study provide a promising solution by avoiding the risk of microbial DNA fragments being amplified by PCR and are suitable for identifying highly degraded materials due to their 200-bp target fragment length. The mini-barcodes are especially suitable for massively parallel sequencing platforms for large-scale identification and monitoring of seedlings [23], thereby enabling the relocation of Pc and Py to their appropriate habitats, enhancing herb quality, and ultimately boosting farmer income.

The potential for hybridization between Pc and Py, as well as with other closely related *Paris* species, remains largely unexplored and warrants future investigation. Given the complex phylogenetic relationships within the *Paris* genus and their significant ecological niche differentiation, natural hybridization events could significantly impact individual environmental adaptability and subsequently influence cultivation practices. Understanding the reproductive compatibility and potential gene flow between species would be crucial for developing appropriate cultivation strategies and maintaining genetic integrity in both wild and cultivated populations [73, 74].

### Feasibility of EM predictions

SDMs are powerful tools essential for studying how environmental changes impact species distributions. Over the past several decades, SDMs have become increasingly important in fields such as biogeography, conservation biology, ecology, and wildlife management. Araújo and colleagues identified five critical challenges for SDMs, including model selection and evaluation [75], highlighting the crucial role models play in predictive outcomes. Traditionally, single models have been used for distribution simulations, with Maxent being the most popular choice; indeed, searching for “maxent” in Google Scholar returns approximately 81,000 results. Using single models is more user-friendly and less resource-intensive than using multiple models, making them attractive options for researchers. However, they often result in predictions that are either under or over-fitted. EMs, despite requiring more sophisticated software and higher learning effort, can avoid or reduce these issues [36]. For example, in this study, the species Pc was evaluated using Maxent, which failed to achieve a True Skill Statistic (TSS) greater than 0.8, indicating that it was not superior to other models. Relying solely on Maxent could thus have compromised the predictive reliability of the present work. Consequently, current research trends favor the development of EMs to more accurately predict suitable habitats for species [37, 65, 76]. Meanwhile, even with EM prediction, SDMs still have some limitations. As mentioned in Araújo et al.’s five challenges, building models with unbiased sampling data remains difficult [59]. Occurrence data are typically sourced from public databases such as CVH and GBIF, which predominantly cover accessible areas like roadsides and forest edges. Consequently, data from remote distribution points are lacking, leading to discrepancies between current data and actual species distributions, and thereby affecting the accuracy of predictions [77]. Furthermore, the phylogenetic analysis based on germplasm genetic data may introduce potential biases in species delimitation. Limited sampling of genetic material, particularly from geographically restricted populations, could result in incomplete representation of intraspecific genetic diversity. The choice of molecular markers and sequencing depth may also influence phylogenetic reconstructions and subsequent taxonomic interpretations, which could affect how occurrence data are assigned to species in SDM analyses.

Additionally, SDMs assume species are in equilibrium with their environment and that current climate-species relationships will remain stable under future conditions—assumptions that may not hold true for all species or scenarios. Climate change may alter species’ physiological tolerances or ecological interactions in ways not captured by correlative models, potentially affecting the reliability of long-term projections [78]. However, these limitations are increasingly being addressed through advances in both modeling approaches and data collection technologies. The integration of mechanistic models that incorporate physiological processes can help overcome equilibrium assumptions, while improved remote sensing and drone technologies facilitate more comprehensive occurrence data collection [79, 80]. These methodological and technological advances collectively promise to enhance the accuracy and reliability of SDM predictions under changing climatic conditions.

### Dominant environmental factors

Temperature and precipitation are regarded as two of the main variables restricting terrestrial plant distributions [81]. In addition, human activity and topography independently affect these distributions [82]. We therefore predicted the potential geographic distributions of Pc and Py by EM using the variables of temperature, precipitation, elevation, and HFI to ensure accuracy and robustness. Different species exhibit varied responses to climate change. The results derived from our biomod2 analysis indicate that climatic factors, specifically temperature and precipitation, significantly influence the habitat ranges of both species compared to topographic and anthropogenic factors. Annual precipitation (Bio 12) was the primary environmental factor affecting Pc, while the temperature annual range (Bio 7) was dominant for Py. This suggests that Py is more sensitive to temperature changes, whereas Pc is more responsive to precipitation. Generally, Pc thrives in environments with lower mean diurnal temperature ranges and altitudes and demonstrates higher resistance to heat and moisture. In contrast, Py is better adapted to withstand cold and drought and prospers in conditions with significant diurnal temperature and precipitation changes (Fig. 2C). These differences likely stem from their early evolutionary divergence (Fig. 1) and subsequent independent evolutionary processes, which led to distinct environmental adaptations and different climatic requirements. Favorable conditions yield quantity, while adversity produces medicinal quality; the harsher growing conditions for Py may explain why its herbal quality is traditionally considered superior to that of Pc [6]. Harsh conditions can increase the accumulation of secondary metabolites, thereby improving herbal quality [83]. For example, low temperature promotes the accumulation of polysaccharides, total alkaloids, and total flavonoids in *Dendrobium officinale* [84], while drought promotes the synthesis of glycyrrhizin by upregulating the glycyrrhizin biosynthesis pathway of *Glycyrrhiza glabra* [85]. These insights can guide the development of efficient cultivation strategies and site selection for these species.

### Impacts of human activity and conservation

Anthropogenic drivers of global change, such as rising greenhouse gas emissions, climate change, biotic invasions, and land-use changes, profoundly affect species distributions [86]. In terms of variable contribution, human activity was the second most important variable shaping the suitable distribution area of Pc, but exhibited a much lower effect on Py. Anthropogenic-induced shifts in ecological niches were also greater in Pc than in Py. Therefore, analyzing ecological niche overlap can effectively quantify these impacts. Overall, human activity had a varied influence on the distribution of these species, with a more substantial impact on Pc. Although there was no significant difference between the impacts of human activity on the two species (Fig. 2C), in terms of extent, human activity was slightly higher in the distribution range of Pc compared to that of Py. The intensity of urbanization and land use in the Yangtze River basin and southern China could partly explain the increased effect on Pc habitats, making human activity the second most important factor influencing its distribution. Human activity is reshaping biological communities and affecting ecosystems [87]. Many studies have highlighted the negative impacts of human activity on biodiversity [87–89]. Balancing the requirements for conserving and sustainably utilizing Traditional Chinese Medicine resources necessitates mitigating anthropogenic impacts and rediscovering a harmonious coexistence between human activity and the sustainable use of other species [90].

Given the endangered status of Pc and Py and their critical importance in Traditional Chinese Medicine, our findings have significant conservation and management implications. First, priority conservation areas should be established in regions with high habitat suitability and low human disturbance, with particular emphasis on areas around Honghe Hani and Yi Autonomous Prefecture and Kunming city for potential germplasm conservation gardens, where long-term suitable habitats overlap for both species. Second, habitat restoration programs should prioritize areas where suitable climate conditions persist but human activities have degraded the habitat quality. Third, guided by the identified suitable cultivation areas and efficient species identification methods, scientifically planned expansion of cultivation zones for both species should be implemented to guarantee high-quality sustainable harvesting while serving as an alternative conservation strategy for wild populations.

### Suitable distribution dynamics and long-term suitable areas

Utilizing projections from global climate models to predict changes in climatically suitable habitats for plant species under various climate-change scenarios effectively illustrates plants’ responses to today’s rapid global changes [86]. Tracking these dynamics helps identify long-term suitable areas, guiding scientific cultivation in the future. In our study, we visualized human impacts on the suitable ranges of the two species. Among areas with a suitability greater than 0.6, Pc’s range contracted by 30%, whereas Py’s range decreased by only 7.5% (Fig. 3). Notably, the Yangtze River basin experienced significant shrinkage for Pc, likely due to increased land use, coinciding with substantial farmland expansion over the past two decades [91]. This farmland expansion is driven by the need to support urban food demands.

Defining long-term suitable planting areas, and thus guiding the selection of future planting sites, was an important objective of this study. However, global warming has dramatically influenced species’ distributions. Our study projected future potential habitats for the two study species under two emission scenarios for the 2050s and 2090s. The future distribution dynamics, particularly under greenhouse gas emissions, are substantial. By the century’s end, Pc’s distribution center is predicted to shift northwestward, while Py’s should remain relatively stable, with minimal geographic migration. In the low-emission scenario (SSP126), Pc benefits from a rapid expansion of ultrahigh-suitability areas, while ultrahigh-suitability areas for Py are maintained, avoiding the potential losses predicted in the high-emission model (SSP585). Meanwhile, estimation of the projected reduction in income per capita from changes in all climatic variables based on empirical models of climate shows that by the end of the century, per capita income in Southeast/East Asia will be reduced by more than 50% if emissions are in the SSP585 scenario [92]. This underscores the need for strict greenhouse gas regulations to ensure the sustainable use of both study species and the protection of economic stability in China and globally. To handle extreme scenarios, we identified and consolidated high-suitability areas (> 0.6) across all future time periods and emission patterns, ultimately delineating long-term viable habitats (Fig. 5). Cultivating these plants in optimized areas, particularly those with extended growing periods, can guarantee a sustainable medicinal supply, enhancing their quality and potency, which are vital for their efficacy in traditional medicine. This approach can strategically inform the layout and development of related industries, optimizing investment outcomes.

## Conclusion

In this study, we reconsidered the taxonomic status of the source taxa of Rhizoma Paridis based on a phylogenomic analysis. Unexpectedly, the two varieties—*P. polyphylla* var. *chinensis* (Pc) and *P. polyphylla* var. *yunnanensis* (Py)—are not closely related and have been diverging for approximately 3.28 million years. They are better treated as distinct species, i.e., *P. chinensis* (Pc) and *P. yunnanensis* (Py). This study further supported the taxonomic revision through phylogenomic evidence and significant ecological niche differentiation, demonstrating the essential role of integrating evolutionary and ecological approaches in accurate species delimitation. To discriminate seeds, seedlings, and processed Rhizoma Paridis of the different source taxa, DNA mini-barcodes were developed based on variations in the plastid genomes of the two species derived from germplasm genetic analysis.

We conducted a robust assessment of the potential distribution of Pc and Py under climate scenarios and human activity by using EM. Environmental factors shaped the distribution range, with annual precipitation identified as the dominant impact factor for Pc and temperature as the dominant impact factor for Py. From the three dimensions of factor contribution rate, ecological niches, and distribution dynamics, we quantified the effects of anthropogenic activity, which affected the distribution range of Pc to a greater extent than it affected Py, which may be attributed to the unevenness of the urbanization process. Under anthropogenic disturbance, Pc showed ultrahigh-suitability areas mainly distributed across Guangxi, Guangdong, Fujian and Taiwan provinces, while Py’s ultrahigh-suitability areas were primarily concentrated in Yunnan province. The suitable areas and ecological conditions for these two species differ significantly. Additionally, a rapid increase in global warming arising from greenhouse gas emissions has significantly affected the distribution of species. Shifts in suitable area under different emission scenarios delineate long-term suitable areas and also show the importance of low-emission scenarios for species survival and sustainable use. Based on our findings, we propose the following actionable recommendations for conservation and cultivation:

For conservation: (1) Germplasm conservation gardens for both species should be established in areas where long-term suitable habitats overlap, particularly around Honghe and Kunming regions; (2) Habitat restoration programs should prioritize high-suitability areas degraded by human activities; (3) Scientific expansion of cultivation areas in high suitability regions could serve as indirect protection for wild resources.

For cultivation: (1) Given the significant ecological niche differentiation between the two species, germplasm identification should be conducted before cultivation when possible; (2) Cultivation site selection should prioritize precipitation considerations for Pc and temperature factors for Py; (3) Long-term cultivation should focus on the stable suitable areas identified in this study.

Overall, efficient species-identification tools, clear habitat dynamics, and long-term planting region delineation will effectively promote the improvement of herbal yield and quality while supporting evidence-based conservation strategies for both wild and cultivated populations. 

## Supplementary Information

Supplementary Material 1. Fig S1. Morphological features of Pc and Py seedlings and sequence data of species-specific sites. Only five accessions of each species are shown. The white bar is 0.5 cm.

Supplementary Material 2. Fig S2. The IQtree of *Pairs* based on dataset II. Numbers on branches indicates the bootstrap value. Branches without labeled bootstrap values indicate a value of 100.

Supplementary Material 3. Fig S3. Evaluation indices of single predictive models.

Supplementary Material 4. Fig S4. The ecological niche overlap of Pc and Py. (A) The green and pink shadings represent densities of species occurrences, blue represents overlap. (B) Correlation and contribution of each variable to the first two components of the PCA-env. (C) histograms of niche equivalency distributions, diamond lines represent observed values. p-value = 1 indicating statistically significant niche differentiation.

Supplementary Material 5. Fig S5. The shift of potential geographical distributions’ centers for Pc and Py under different time periods and emission scenarios. GZ, Guizhou; HN, Hunan; GX, Guangxi; SC, Sichuan; YN, Yunnan.

Supplementary Material 6. Table S1. Detail information of self-sequenced or downloaded plastome in this study.

Supplementary Material 7. Table S2. Seedling information of Pc and Py used in this study.

Supplementary Material 8. Table S3. The description of selected climatic variables.

Supplementary Material 9. Table S4. Detailed single model information for ensemble prediction used.

Supplementary Material 10. Table S5. The detail information of species-specific sites.

Supplementary Material 11. Table S6. The suitable cultivation area for Pc and Py under different scenarios and time periods.

## Data Availability

The newly sequenced data were deposited in the GenBank (https://www.ncbi.nlm.nih.gov) with accession number: PQ399717- PQ399719. In addition, details of the previously published sequences used in this study are provided in Table S1. Clinical trial number: not applicable.

## References

[1] Zhao G, Cui X, Sun J, Li T, Wang Q, Ye X, Fan B. Analysis of the distribution pattern of Chinese Ziziphus Jujuba under climate change based on optimized biomod2 and maxent models. Ecol Ind. 2021;132:108256.

[;2] Allen M, Dube O, Solecki W, Aragón-Durand F, Cramer W, Humphreys S, Kainuma M. Special report: Global warming of 1.5 C. Intergovernmental Panel on Climate Change. 2018;1(5):43–50.

[3] Xu W-B, Svenning J-C, Chen G-K, Zhang M-G, Huang J-H, Chen B, Ordonez A, Ma K-P. Human activities have opposing effects on distributions of narrow-ranged and widespread plant species in China. Proc Natl Acad Sci. 2019;116(52):26674–81. 31843905 10.1073/pnas.1911851116 PMC6936463

[4] Li Y, Kong D, Fu Y, Sussman MR, Wu H. The effect of developmental and environmental factors on secondary metabolites in medicinal plants. Plant Physiol Biochem. 2020;148:80–9. 31951944 10.1016/j.plaphy.2020.01.006

[5] Lin X, Yin J, Wang Y, Yao J, Li QQ, Latzel V, Bossdorf O, Zhang Y-Y. Environment-induced heritable variations are common in Arabidopsis Thaliana. Nat Commun. 2024;15(1):4615. 38816460 10.1038/s41467-024-49024-3 PMC11139905

[6] Wang J, Yang Q, Jiang Y, Shang M, Yang Y, Duan B. Geo-herbalism study of Paridis rhizoma. Chin Traditional Herb Drugs. 2022;53(8):2572–81.

[7] Yan X, Wang D, Zhang A, Xia J, Jiao J, Ghanim M, Xiaokun O, He X, Shi R. Understory growth of Paris polyphylla accumulates a reservoir of secondary metabolites of plants. Front Microbiol. 2024;15:1400616. 39473849 10.3389/fmicb.2024.1400616 PMC11518744

[8] Deb CR. Studies on vegetative and reproductive ecology of Paris polyphylla smith: a vulnerable medicinal plant. Am J Plant Sci. 2015;06(16):2561–8.

[9] Paul A, GAJUREL PR, DAS AK. Threats and conservation of Paris polyphylla an endangered, highly exploited medicinal plant in the Indian Himalayan region. Biodiversitas J Biol Divers. 2015;16(2):295–302.

[10] Thakur U, Shashni S, Thakur N, Rana SK, Singh A. A review on Paris polyphylla smith: a vulnerable medicinal plant species of a global significance. J Appl Res MedicinalAromatic Plants. 2023;33:100447.

[11] Thapa CB, Paudel MR, Bhattarai HD, Pant KK, Devkota HP, Adhikari YP, Pant B. Bioactive secondary metabolites in Paris polyphylla Sm. and their biological activities: a review. Heliyon. 2022;8(2):e08982. 35243100 10.1016/j.heliyon.2022.e08982 PMC8881664

[12] Hebert PD, Cywinska A, Ball SL, DeWaard JR. Biological identifications through DNA barcodes. Proceedings of the Royal Society of London Series B: Biological Sciences. 2003;270(1512):313–321. 10.1098/rspb.2002.2218 PMC1691236 12614582

[13] Hollingsworth PM, Graham SW, Little DP. Choosing and using a plant DNA barcode. PLoS ONE. 2011;6(5):e19254. 21637336 10.1371/journal.pone.0019254 PMC3102656

[14] Cheng T, Xu C, Lei L, Li C, Zhang Y, Zhou S. Barcoding the Kingdom plantae: new PCR primers for ITS regions of plants with improved universality and specificity. Mol Ecol Resour. 2016;16(1):138–49. 26084789 10.1111/1755-0998.12438

[15] Dong W, Cheng T, Li C, Xu C, Long P, Chen C, Zhou S. Discriminating plants using the DNA barcode rbclb: an appraisal based on a large data set. Mol Ecol Resour. 2014;14(2):336–43. 24119263 10.1111/1755-0998.12185

[16] Dong W, Xu C, Li C, Sun J, Zuo Y, Shi S, Cheng T, Guo J, Zhou S. ycf1, the most promising plastid DNA barcode of land plants. Sci Rep. 2015;5:8348. 25672218 10.1038/srep08348 PMC4325322

[17] Liu Y, Ding K, Liang L, Zhang Z, Chen K. Comparative study on Chloroplast genome of tamarix species. Ecol Evol. 2024;14(10):e70353. 39360124 10.1002/ece3.70353 PMC11445282

[18] Miao K, Wang T, Tang L, Hou L, Ji Y. Establishing the first reference library for utilizing high-throughput sequencing technologies in identifying medicinal and endangered Paris species (Melanthiaceae). Industrial Crops Prod. 2024;218:118871.

[19] Zhang W, Sun Y, Liu J, Xu C, Zou X, Chen X, Liu Y, Wu P, Yang X, Zhou S. DNA barcoding of oryza: conventional, specific, and super barcodes. Plant Mol Biol. 2021;105:215–28. 32880855 10.1007/s11103-020-01054-3 PMC7858216

[20] Xu C, Dong W, Shi S, Cheng T, Li C, Liu Y, Wu P, Wu H, Gao P, Zhou S. Accelerating plant DNA barcode reference library construction using herbarium specimens: improved experimental techniques. Mol Ecol Resour. 2015;15(6):1366–74. 25865498 10.1111/1755-0998.12413

[21] Dong W, Liu H, Xu C, Zuo Y, Chen Z, Zhou S. A Chloroplast genomic strategy for designing taxon specific DNA mini-barcodes: a case study on ginsengs. BMC Genet. 2014;15:138. 25526752 10.1186/s12863-014-0138-z PMC4293818

[22] Meusnier I, Singer GA, Landry JF, Hickey DA, Hebert PD, Hajibabaei M. A universal DNA mini-barcode for biodiversity analysis. BMC Genomics. 2008;9:214. 18474098 10.1186/1471-2164-9-214 PMC2396642

[23] Wang Y, Sun J, Zhao Z, Xu C, Qiao P, Wang S, Wang M, Xu Z, Yuan Q, Guo L, et al. Multiplexed massively parallel sequencing of plastomes provides insights into the genetic diversity, population structure, and phylogeography of wild and cultivated Coptis chinensis. Front Plant Sci. 2022;13:923600. 35873994 10.3389/fpls.2022.923600 PMC9302112

[24] Shao L, Qiao P, Wang J, Peng Y, Wang Y, Dong W, Li J. Comparative analysis of jujube and sour jujube gave insight into their difference in genetic diversity and suitable habitat. 2024;15:1322285. 10.3389/fgene.2024.1322285 PMC10878421 38380425

[25] Wang D, Shi C, Alamgir K, Kwon S, Pan L, Zhu Y, Yang X. Global assessment of the distribution and conservation status of a key medicinal plant (Artemisia annua L.): the roles of climate and anthropogenic activities. Sci Total Environ. 2022;821:153378. 35085641 10.1016/j.scitotenv.2022.153378

[26] Zhang W, Chen X, Liu R, Song X, Liu G, Zou J, Qian Z, Zhu Z, Cui L. Realized niche shift associated with Galinsoga quadriradiata (Asteraceae) invasion in China. J Plant Ecol. 2022;15(3):538–48.

[27] Wang Y, Wang J, Garran TA, Liu H, Lin H, Luo J, Yuan Q, Sun J, Dong W, Guo L. Genetic diversity and population divergence OfLeonurus Japonicusand its distribution dynamic changes from the last interglacial to the present in China. BMC Plant Biol. 2023;23(1):276. 37226102 10.1186/s12870-023-04284-x PMC10210291

[28] Phillips SJ, Dudík M. Modeling of species distributions with maxent: new extensions and a comprehensive evaluation. J Ecography. 2008;31(2):161–75.

[29] Nelder JA, Wedderburn RW. Generalized linear models. J Royal Stat Soc Ser A: Stat Soc. 1972;135(3):370–84.

[30] Friedman JH. Multivariate adaptive regression splines. Annals Stat. 1991;19(1):1–67.

[31] Booth TH, Nix HA, Busby JR, Hutchinson MF. BIOCLIM: the first species distribution modelling package, its early applications and relevance to most current MAXENT studies. Divers Distrib. 2014;20(1):1–9.

[32] Hirzel AH, Hausser J, Chessel D, Perrin NJE. Ecological-niche factor analysis: how to compute habitat‐suitability maps without absence data? Ecology. 2002;83(7):2027–36.

[33] Lopatin J, Dolos K, Hernández H, Galleguillos M, Fassnacht F. Comparing generalized linear models and random forest to model vascular plant species richness using lidar data in a natural forest in central Chile. Remote Sens Environ. 2016;173:200–10.

[34] Hao T, Elith J, Guillera-Arroita G, Lahoz‐Monfort JJ. A review of evidence about use and performance of species distribution modelling ensembles like BIOMOD. Divers Distrib. 2019;25(5):839–52.

[35] Dormann CF, Calabrese JM, Guillera-Arroita G, et al Model averaging in ecology: a review of Bayesian, information‐theoretic, and tactical approaches for predictive inference. Ecol Monogr. 2018;88(4):485–504. 10.1002/ecm.1309.

[36] Xian X, Zhao H, Wang R, Huang H, Chen B, Zhang G, Liu W, Wan F. Climate change has increased the global threats posed by three ragweeds (Ambrosia L.) in the anthropocene. Sci Total Environ. 2023;859:160252. 36427731 10.1016/j.scitotenv.2022.160252

[37] Fang Y, Zhang X, Wei H, Wang D, Chen R, Wang L, Gu W. Predicting the invasive trend of exotic plants in China based on the ensemble model under climate change: A case for three invasive plants of Asteraceae. Sci Total Environ. 2021;756:143841. 33248784 10.1016/j.scitotenv.2020.143841

[38] Ardestani EG, Ghahfarrokhi ZH. Ensembpecies distribution modeling of salvia hydrangea under future climate change scenarios in central Zagros Mountains, Iran. Global Ecol Conserv. 2021;26:e01488.

[39] Di Cola V, Broennimann O, Petitpierre B, Breiner FT, d’Amen M, Randin C, Engler R, Pottier J, Pio D, Dubuis A. Ecospat: an R package to support Spatial analyses and modeling of species niches and distributions. Ecography. 2017;40(6):774–87.

[40] Li J, Wang S, Yu J, Wang L, Zhou S. A modified CTAB protocol for plant DNA extraction. Chin Bull Bot. 2013;48(1):72–8.

[41] Chen S. Ultrafast one-pass FASTQ data preprocessing, quality control, and deduplication using Fastp. Imeta. 2023;2(2):e107. 38868435 10.1002/imt2.107 PMC10989850

[42] Jin J, Yu W, Yang J, Song Y, dePamphilis CW, Yi T, Li D. GetOrganelle: a fast and versatile toolkit for accurate de Novo assembly of organelle genomes. Genome Biol. 2020;21(1):241. 32912315 10.1186/s13059-020-02154-5 PMC7488116

[43] Shi L, Chen H, Jiang M, Wang L, Wu X, Huang L, Liu C. CPGAVAS2, an integrated plastome sequence annotator and analyzer. Nucleic Acids Res. 2019;47(W1):W65–73. 31066451 10.1093/nar/gkz345 PMC6602467

[44] Katoh K, Rozewicki J, Yamada KD. MAFFT online service: multiple sequence alignment, interactive sequence choice and visualization. Brief Bioinform. 2019;20(4):1160–6. 28968734 10.1093/bib/bbx108 PMC6781576

[45] Lyu F, Han F, Ge C, Mao W, Chen L, Hu H, Chen G, Lang Q, Fang C. OmicStudio: a composable bioinformatics cloud platform with real-time feedback that can generate high‐quality graphs for publication. Imeta. 2023;2(1):e85. 38868333 10.1002/imt2.85 PMC10989813

[46] Zhou L, Feng T, Xu S, Gao F, Lam TT, Wang Q, Wu T, Huang H, Zhan L, Li L, et al. Ggmsa: a visual exploration tool for multiple sequence alignment and associated data. Brief Bioinform. 2022;23(4):bbac222. 35671504 10.1093/bib/bbac222

[47] Castresana J. GBLOCLKS: selection of conserved blocks from multiple alignments for their use in phylogenetic analysis. Version 0.91b. Mol Biol Evol. 2000;17(4):540–52. 10742046 10.1093/oxfordjournals.molbev.a026334

[48] Zhang D, Gao F, Jakovlic I, Zou H, Zhang J, Li W, Wang G. PhyloSuite: an integrated and scalable desktop platform for streamlined molecular sequence data management and evolutionary phylogenetics studies. Mol Ecol Resour. 2020;20(1):348–55. 31599058 10.1111/1755-0998.13096

[49] Nguyen LT, Schmidt HA, von Haeseler A, Minh BQ. IQ-TREE: a fast and effective stochastic algorithm for estimating maximum-likelihood phylogenies. Mol Biol Evol. 2015;32(1):268–74. 25371430 10.1093/molbev/msu300 PMC4271533

[50] Bouckaert R, Vaughan TG, Barido Sottani J, Duchene S, Fourment M, Gavryushkina A, Heled J, Jones G, Kuhnert D, De Maio N, et al. BEAST 2.5: an advanced software platform for bayesian evolutionary analysis. PLoS Comput Biol. 2019;15(4):e1006650. 30958812 10.1371/journal.pcbi.1006650 PMC6472827

[51] Yang L, Yang Z, Liu C, He Z, Zhang Z, Yang J, Liu H, Yang J, Ji Y. Chloroplast phylogenomic analysis provides insights into the evolution of the largest eukaryotic genome holder. BMC Plant Biol. 2019;19(1):293. 31272375 10.1186/s12870-019-1879-7 PMC6611055

[52] Ji Y, Yang L, Chase MW, Liu C, Yang Z, Yang J, Yang J-B, Yi T-S. Plastome phylogenomics, biogeography, and clade diversification of Paris (Melanthiaceae). BMC Plant Biol. 2019;19:543. 31805856 10.1186/s12870-019-2147-6 PMC6896732

[53] Brown J, Bennett J, French C. SDMtoolbox 2.0: the next generation Python-based GIS toolkit for landscape genetic, biogeographic and species distribution model analyses. PeerJ. 2017;5:e4095. 29230356 10.7717/peerj.4095 PMC5721907

[54] Amatulli G, Domisch S, Tuanmu MN, Parmentier B, Ranipeta A, Malczyk J, Jetz W. A suite of global, cross-scale topographic variables for environmental and biodiversity modeling. Sci Data. 2018;5:180040. 29557978 10.1038/sdata.2018.40 PMC5859920

[55] Venter O, Sanderson EW, Magrach A, Allan JR, Beher J, Jones KR, Possingham HP, Laurance WF, Wood P, Fekete BM, et al. Last of the wild Project, version 3 (LWP-3): 2009 human Footprint, 2018 Release. In. Palisades. New York: NASA Socioeconomic Data and Applications Center (SEDAC); 2018.

[56] Dong X, Chu YM, Gu X, Huang Q, Zhang J, Bai W. Suitable habitat prediction of Sichuan snub-nosed monkeys (Rhinopithecus roxellana) and its implications for conservation in Baihe nature Reserve, Sichuan, China. Environ Sci Pollut Res Int. 2019;26(31):32374–84. 31602599 10.1007/s11356-019-06369-3

[57] Valentin S, Levin S. Plotbiomes: Plot Whittaker biomes with ggplot2. R package version 0.0.0.9001.2021. https://github.com/valentinitnelav/plotbiomes

[58] Jaureguiberry P, Titeux N, Wiemers M, Bowler DE, Coscieme L, Golden AS, Guerra CA, Jacob U, Takahashi Y, Settele J. The direct drivers of recent global anthropogenic biodiversity loss. Sci Adv. 2022;8(45):eabm9982. 36351024 10.1126/sciadv.abm9982 PMC9645725

[59] Pecl GT, Araújo MB, Bell JD, Blanchard J, Bonebrake TC, Chen I-C, Clark TD, Colwell RK, Danielsen F, Evengård B. Biodiversity redistribution under climate change: impacts on ecosystems and human well-being. Science. 2017;355(6332):eaai9214. 28360268 10.1126/science.aai9214

[60] Shaw RG, Etterson JR. Rapid climate change and the rate of adaptation: insight from experimental quantitative genetics. New Phytol. 2012;195(4):752–65. 22816320 10.1111/j.1469-8137.2012.04230.x

[61] Parmesan C. Ecological and evolutionary responses to recent climate change. Annu Rev Ecol Evol Syst. 2006;37(1):637–69.

[62] Guéguen MBH, Thuiller W. biomod2: Ensemble Platform for Species Distribution Modeling. R package version 4.3-2-3. 2025. https://biomodhub.github.io/biomod2/.

[63] Bebber DP, Field E, Gui H, Mortimer P, Holmes T, Gurr SJ. Many unreported crop pests and pathogens are probably already present. Glob Chang Biol. 2019;25(8):2703–13. 31237022 10.1111/gcb.14698

[64] Changjun G, Yanli T, Linshan L, Bo W, Yili Z, Haibin Y, Xilong W, Zhuoga Y, Binghua Z, Bohao C. Predicting the potential global distribution of ageratina adenophora under current and future climate change scenarios. Ecol Evol. 2021;11(17):12092–113. 34522363 10.1002/ece3.7974 PMC8427655

[65] Huang D, An Q, Huang S, Tan G, Quan H, Chen Y, Zhou J, Liao H. Biomod2 modeling for predicting the potential ecological distribution of three fritillaria species under climate change. Sci Rep. 2023;13(1):18801. 37914761 10.1038/s41598-023-45887-6 PMC10620159

[66] Warren DL, Glor RE, Turelli M. ENMTools: a toolbox for comparative studies of environmental niche models. Ecography. 2010;33(3):607–11.

[67] Warren DL, Glor RE, Turelli M. Environmental niche equivalency versus conservatism: quantitative approaches to niche evolution. Evolution. 2008;62(11):2868–83. 18752605 10.1111/j.1558-5646.2008.00482.x

[68] Zhou N, Miao K, Hou L, Liu H, Chen J, Ji Y. Phylotranscriptomic analyses reveal the evolutionary complexity of Paris L. (Melanthiaceae), a morphologically distinctive genus with significant pharmaceutical importance. Ann Bot. 2024;134(7):1277–90. 39221840 10.1093/aob/mcae156 PMC11688527

[69] Shang J, Zhao Q, Yan P, Sun M, Sun H, Liang H, Zhang D, Qian Z, Cui L. Environmental factors influencing potential distribution of schisandra Sphenanthera and its accumulation of medicinal components. Front Plant Sci. 2023;14:1302417. 38162305 10.3389/fpls.2023.1302417 PMC10756911

[70] Zhao Y, Zhang J, Yuan T, Shen T, Li W, Yang S, Hou Y, Wang Y, Jin H. Discrimination of wild Paris based on near infrared spectroscopy and high performance liquid chromatography combined with multivariate analysis. PLoS ONE. 2014;9(2):e89100. 24558477 10.1371/journal.pone.0089100 PMC3928364

[71] Yin H, Zhang K, Ren Z, Zhao J, Gao C, Chen R. Paris polyphylla var. Yunnanensis genotype classification and fluorescence visual identification. Agron J. 2022;114(4):1971–80.

[72] Duan B-Z, Wang Y-P, Fang H-L, Xiong C, Li X-W, Wang P, Chen S-L. Authenticity analyses of rhizoma Paridis using barcoding coupled with high resolution melting (Bar-HRM) analysis to control its quality for medicinal plant product. Chin Med. 2018;13:1–10. 29449876 10.1186/s13020-018-0162-4 PMC5806261

[73] Zhu Z, Wang Y, Liu S, Wang S, Li J, Fang C, Liu Y, Yang X, Tian D, Song S. Genomic atlas of 8,105 accessions reveals stepwise domestication, global dissemination, and improvement trajectories in soybean. Cell. 2025;188(23):6519–35. 41038165 10.1016/j.cell.2025.09.007

[74] Shi Y, Li B, Gao Y, Wang X, Liu Y, Lu X, Lin H, Li W, Lai D, Hao MJGB. Phylogenomics provides comprehensive insights into the evolutionary relationships among cultivated buckwheat species. Genome Biol. 2025;26(1):327. 41035109 10.1186/s13059-025-03793-2 PMC12487180

[75] Araújo MB, Guisan A. Five (or so) challenges for species distribution modelling. J Biogeogr. 2006;33(10):1677–88.

[76] Pacifici K, Reich BJ, Miller DA, Pease BS. Resolving misaligned Spatial data with integrated species distribution models. Ecology. 2019;100(6):e02709. 30933314 10.1002/ecy.2709 PMC6851831

[77] Phillips SJ, Dudik M, Elith J, Graham CH, Lehmann A, Leathwick J, Ferrier S. Sample selection bias and presence-only distribution models: implications for background and pseudo-absence data. Ecol Appl. 2009;19(1):181–97. 19323182 10.1890/07-2153.1

[78] Wiens JA, Stralberg D, Jongsomjit D, Howell CA, Snyder MA. Niches, models, and climate change: assessing the assumptions and uncertainties. Proceedings of the National Academy of Sciences. 2009;106:19729–36. 10.1073/pnas.0901639106 PMC2780938 19822750

[79] Robinson JM, Harrison PA, Mavoa S, Breed MF. Existing and emerging uses of drones in restoration ecology. Methods Ecol Evol. 2022;13(9):1899–911.

[80] Cruzan MB, Weinstein BG, Grasty MR, Kohrn BF, Hendrickson EC, Arredondo TM, Thompson PG. Small unmanned aerial vehicles (micro-UAVs, drones) in plant ecology. Appl Plant Sci. 2016;4(9):1600041. 10.3732/apps.1600041 PMC5033362 27672518

[81] Wu Z, Dijkstra P, Koch GW, Peñuelas J, Hungate BA. Responses of terrestrial ecosystems to temperature and precipitation change: A meta-analysis of experimental manipulation. Glob Change Biol. 2011;17(2):927–42.

[82] González-Moreno P, Diez JM, Ibáñez I, Font X, Vilà M. Plant invasions are context‐dependent: multiscale effects of climate, human activity and habitat. Divers Distrib. 2014;20(6):720–31.

[83] Pant P, Pandey S, Dall’Acqua S. The influence of environmental conditions on secondary metabolites in medicinal plants: A literature review. Chem Biodivers. 2021;18(11):e2100345. 34533273 10.1002/cbdv.202100345

[84] Yuan Y, Tang X, Jia Z, Li C, Ma J, Zhang J. The effects of ecological factors on the main medicinal components of dendrobium officinale under different cultivation modes. Forests. 2020;11(1):94.

[85] Hosseini MS, Samsampour D, Ebrahimi M, Abadía J, Khanahmadi M. Effect of drought stress on growth parameters, osmolyte contents, antioxidant enzymes and glycyrrhizin synthesis in licorice (Glycyrrhiza glabra L.) grown in the field. Phytochemistry. 2018;156:124–34. 30278303 10.1016/j.phytochem.2018.08.018

[86] Franklin J, Serra-Diaz JM, Syphard AD, Regan HM. Global change and terrestrial plant community dynamics. Proc Natl Acad Sci. 2016;113(14):3725–34. 26929338 10.1073/pnas.1519911113 PMC4833242

[87] Bowler DE, Bjorkman AD, Dornelas M, Myers-Smith IH, Navarro LM, Niamir A, Supp SR, Waldock C, Winter M, Vellend M. Mapping human pressures on biodiversity across the planet uncovers anthropogenic threat complexes. People Nat. 2020;2(2):380–94.

[88] Zheng Z, Ma T, Roberts P, Li Z, Yue Y, Peng H, Huang K, Han Z, Wan Q, Zhang Y. Anthropogenic impacts on late holocene land-cover change and floristic biodiversity loss in tropical southeastern Asia. Proc Natl Acad Sci. 2021;118(40):e2022210118. 34580205 10.1073/pnas.2022210118 PMC8501839

[89] Brummitt NA, Bachman SP, Griffiths-Lee J, Lutz M, Moat JF, Farjon A, Donaldson JS, Hilton-Taylor C, Meagher TR, Albuquerque S. Green plants in the red: A baseline global assessment for the IUCN sampled red list index for plants. PLoS ONE. 2015;10(8):e0135152. 26252495 10.1371/journal.pone.0135152 PMC4529080

[90] Storch D, Šímová I, Smyčka J, Bohdalková E, Toszogyova A, Okie JG. Biodiversity dynamics in the anthropocene: how human activities change equilibria of species richness. Ecography. 2022;2022(4):e05778.

[91] Wang J, Lin Y, Zhai T, He T, Qi Y, Jin Z, Cai Y. The role of human activity in decreasing ecologically sound land use in China. Land Degrad Dev. 2018;29(3):446–60.

[92] Kotz M, Levermann A, Wenz L. The economic commitment of climate change. Nature. 2024;628(8008):551–7. 38632481 10.1038/s41586-024-07219-0 PMC11023931

