# Supplementary figures and images for: Assessment of suitable cultivation area for Paris polyphylla var. chinensis and var. yunnanensis under anthropogenic disturbance based on ensemble modeling and germplasm identification
Source: BMC Plant Biol. 2026 Jan 14;26:241. doi: 10.1186/s12870-025-08010-7 (PMC12874964; doi:10.1186/s12870-025-08010-7)

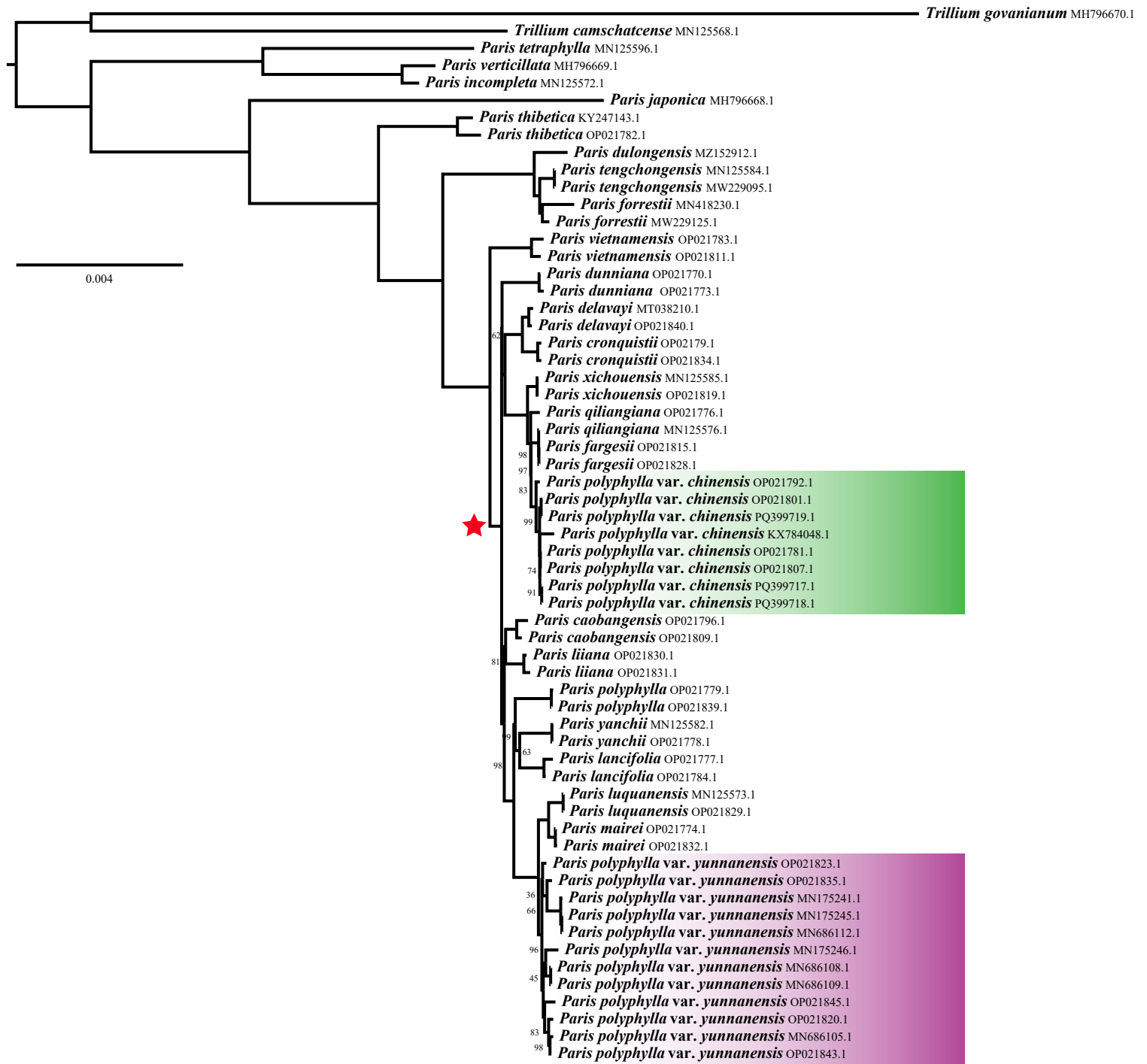

Supplement: Supplementary file 2 — Supplementary Material 2. Fig S2. The IQtree of Pairs based on dataset II. Numbers on branches indicates the bootstrap value. Branches without labeled bootstrap values indicate a value of 100. [file 12870_2025_8010_MOESM2_ESM.pdf]

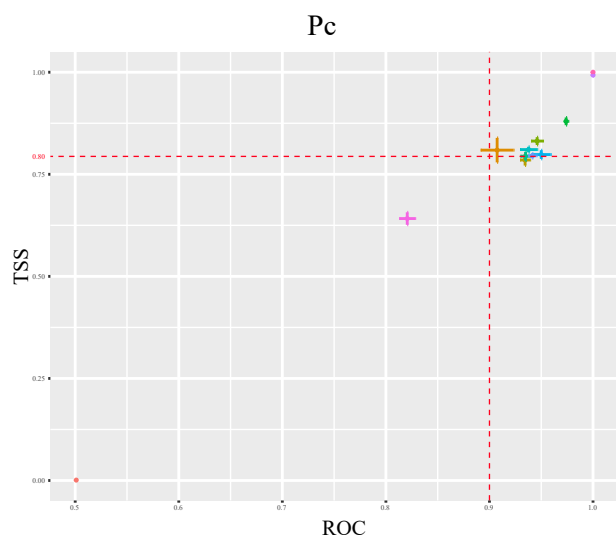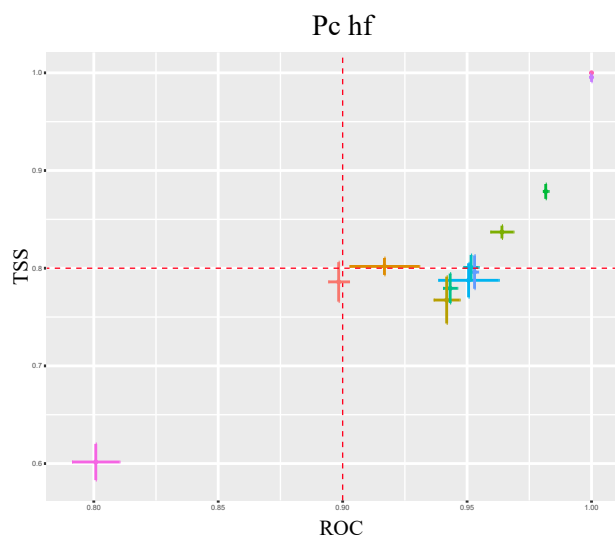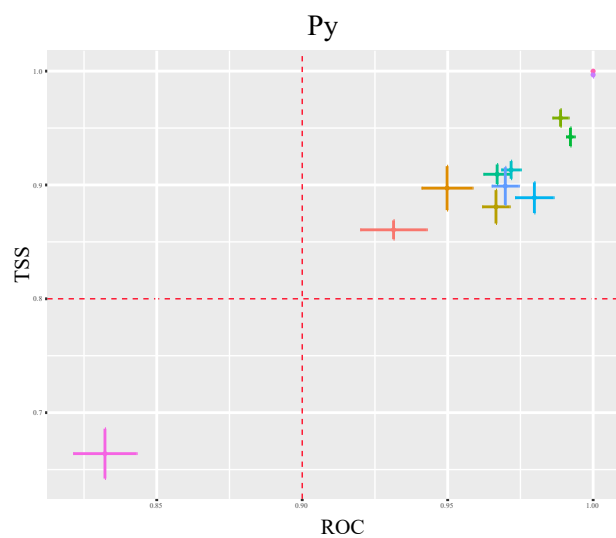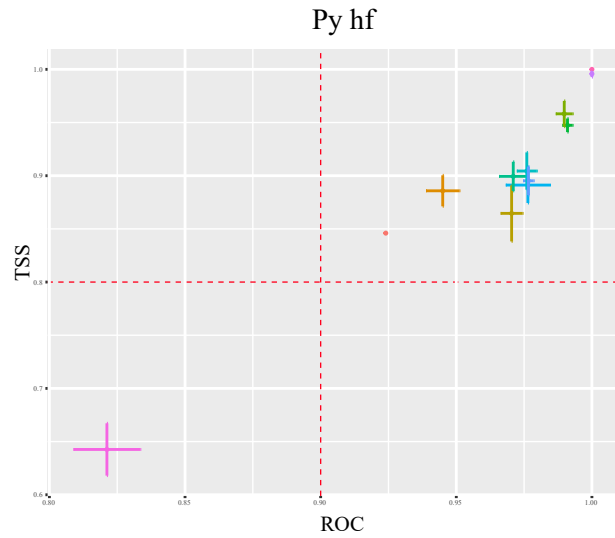

- ANN
- CTA
- FDA
- GAM
- GBM
- GLM
- MARS
- MAXENT
- MAXNET
- RF
- SRE
- XGBOOST

Supplement: Supplementary file 3 — Supplementary Material 3. Fig S3. Evaluation indices of single predictive models. [file 12870_2025_8010_MOESM3_ESM.pdf]

P. c vs P. y Niche Overlap

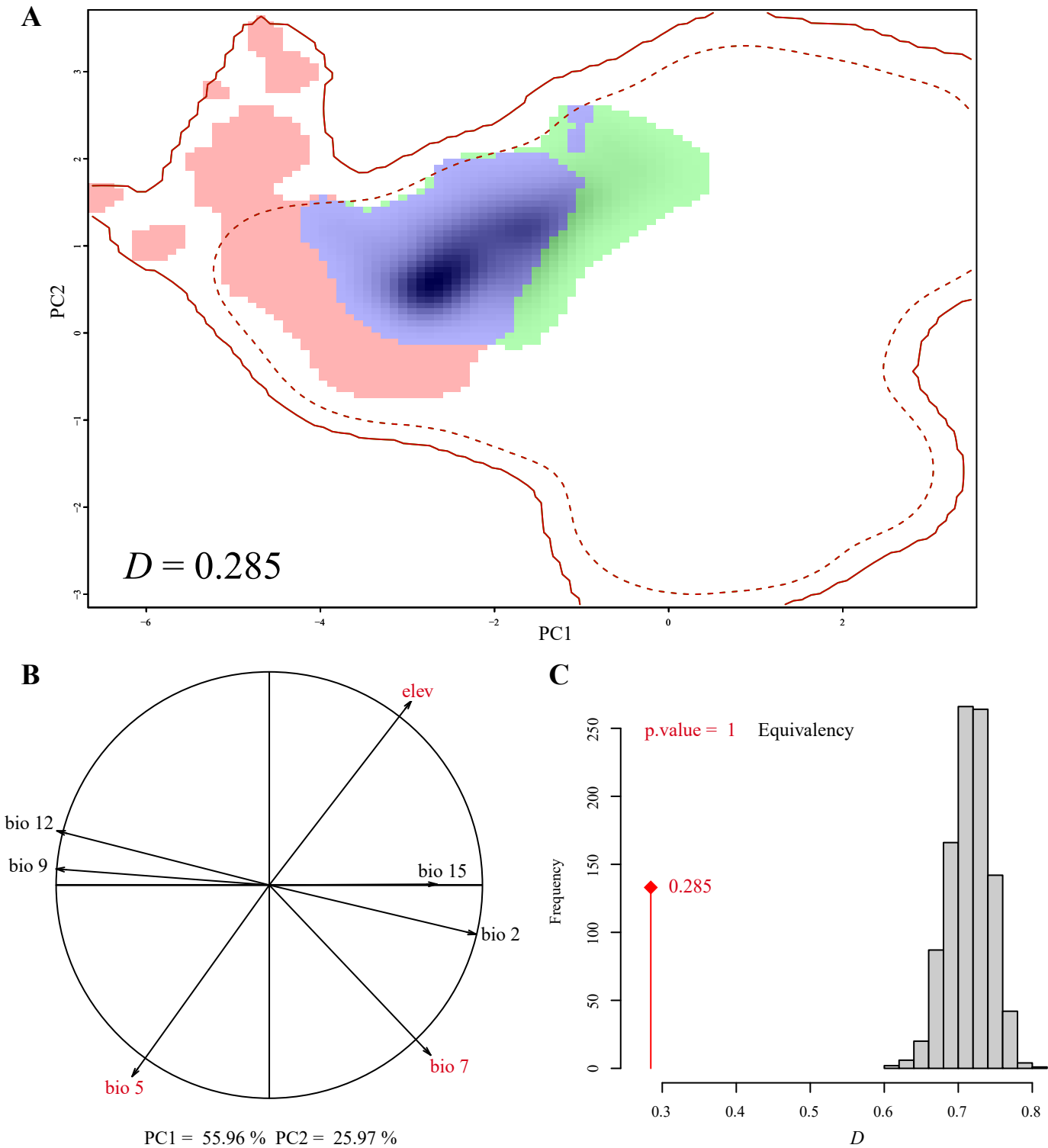

Supplement: Supplementary file 4 — Supplementary Material 4. Fig S4. The ecological niche overlap of Pc and Py. (A) The green and pink shadings represent densities of species occurrences, blue represents overlap. (B) Correlation and contribution of each variable to the first two components of the PCA-env. (C) histograms of niche equivalency distributions, diamond lines represent observed values. p-value = 1 indicating statistically significant niche differentiation. [file 12870_2025_8010_MOESM4_ESM.pdf]

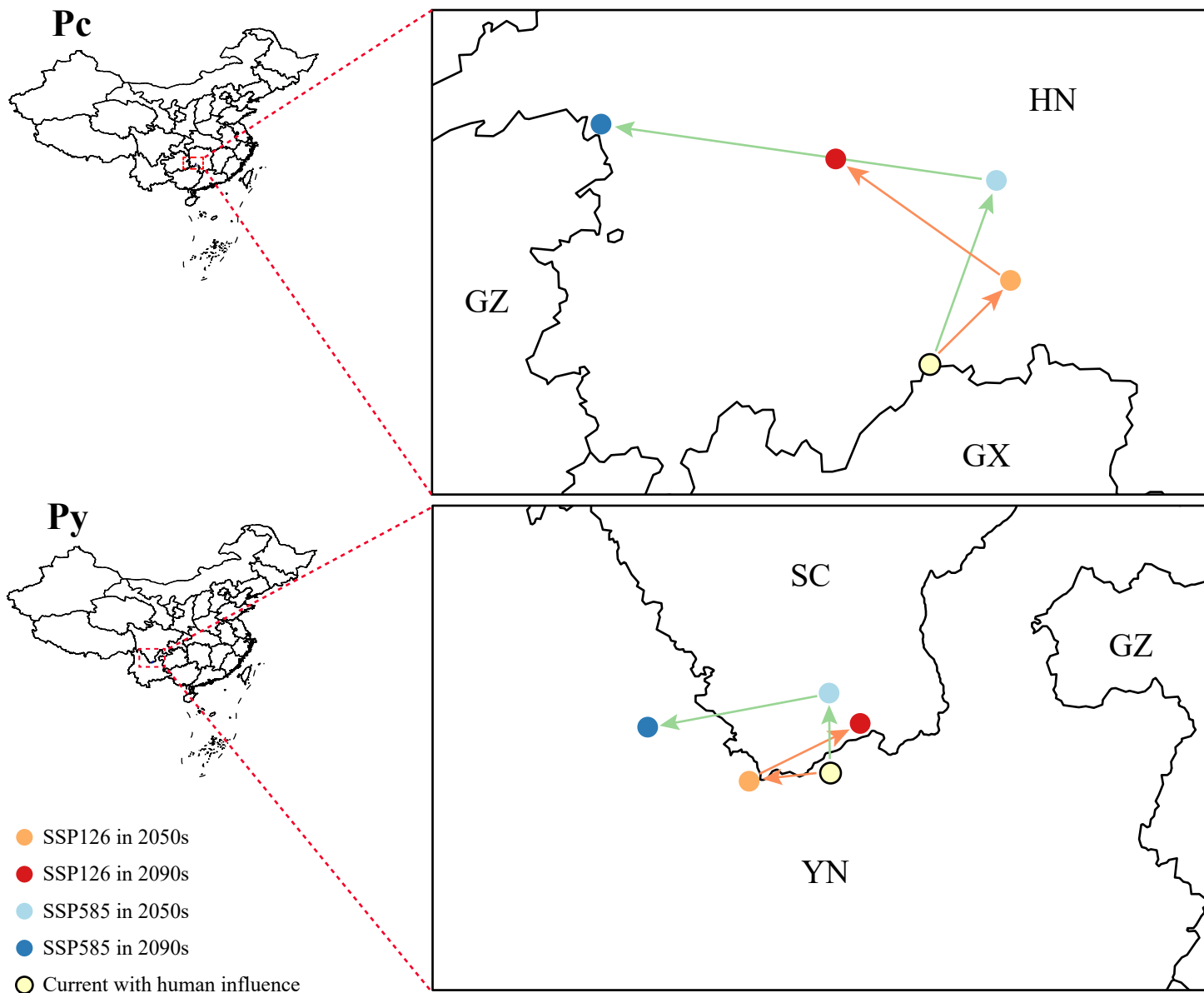

Supplement: Supplementary file 5 — Supplementary Material 5. Fig S5. The shift of potential geographical distributions’ centers for Pc and Py under different time periods and emission scenarios. GZ, Guizhou; HN, Hunan; GX, Guangxi; SC, Sichuan; YN, Yunnan. [file 12870_2025_8010_MOESM5_ESM.pdf]
