# Supplementary material for: Assessment of suitable cultivation area for Paris polyphylla var. chinensis and var. yunnanensis under anthropogenic disturbance based on ensemble modeling and germplasm identification
Source: BMC Plant Biol. 2026 Jan 14;26:241. doi: 10.1186/s12870-025-08010-7 (PMC12874964; doi:10.1186/s12870-025-08010-7)
Supplement: Supplementary file 1 — Supplementary Material 1. Fig S1. Morphological features of Pc and Py seedlings and sequence data of species-specific sites. Only five accessions of each species are shown. The white bar is 0.5 cm. [file 12870_2025_8010_MOESM1_ESM.pdf]

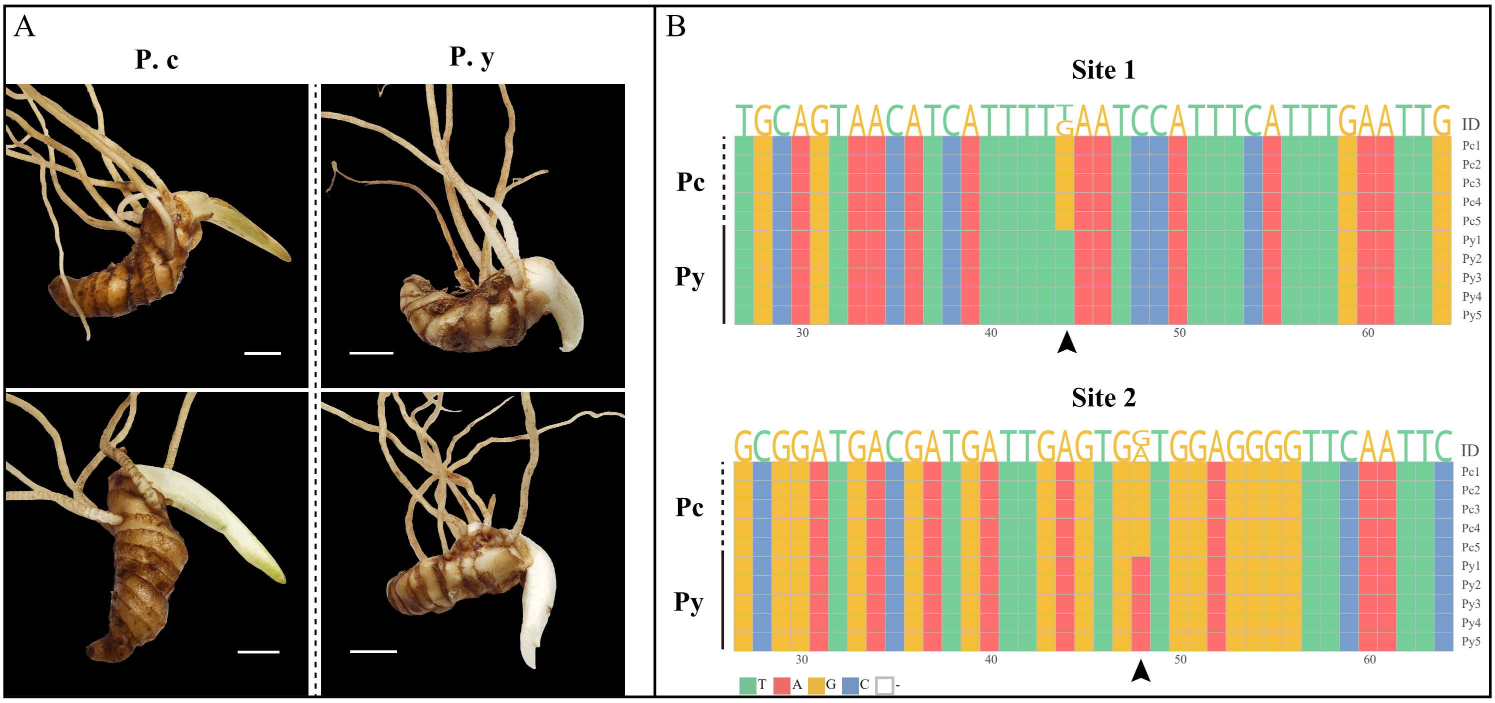
A
P. c
P. y
B
Site 1
ID
Pc
Py
Pc1
Pc2
Pc3
Pc4
Pc5
Py1
Py2
Py3
Py4
Py5
30
40
50
60
Site 2
ID
Pc
Py
Pc1
Pc2
Pc3
Pc4
Pc5
Py1
Py2
Py3
Py4
Py5
30
40
50
60
T
A
G
C
-
